# Genomic divergence and adaptive convergence in *Drosophila simulans* from Evolution Canyon, Israel

**DOI:** 10.1073/pnas.1720938116

**Published:** 2019-05-24

**Authors:** Lin Kang, Eugenia Rashkovetsky, Katarzyna Michalak, Harold R. Garner, James E. Mahaney, Beverly A. Rzigalinski, Abraham Korol, Eviatar Nevo, Pawel Michalak

**Affiliations:** ^a^Edward Via College of Osteopathic Medicine, Blacksburg, VA 24060;; ^b^Institute of Evolution, Haifa University, 3498838 Haifa, Israel;; ^c^Biocomplexity Institute, Virginia Tech, Blacksburg, VA 24061;; ^d^The Gibbs Cancer Center and Research Institute, Spartanburg, SC 29303;; ^e^Center for One Health Research, Virginia-Maryland College of Veterinary Medicine, Blacksburg, VA 24060

**Keywords:** adaptive evolution, selective sweeps, evolutionary convergence, local adaptations

## Abstract

Biodiversity refugia formed by unique features of the Mediterranean arid landscape, such as the dramatic ecological contrast of “Evolution Canyon,” provide a natural laboratory in which local adaptations to divergent microclimate conditions can be investigated. Significant insights have been provided by studies of *Drosophila melanogaster* diversifying along the thermal gradient in Evolution Canyon, but a comparative framework to survey adaptive convergence across sister species at the site has been lacking. To fill this void, we present an analysis of genomic polymorphism and evolutionary divergence of *Drosophila simulans*, a close relative of *Drosophila melanogaster* with which it co-occurs on both slopes of the canyon. Our results show even deeper interslope divergence in *D. simulans* than in *D. melanogaster*, with extensive signatures of selective sweeps present in flies from both slopes but enhanced in the population from the hotter and drier south-facing slope. Interslope divergence was enriched for genes related to electrochemical balance and transmembrane transport, likely in response to increased selection for dehydration resistance on the hotter slope. Both species shared genomic regions that underwent major selective sweeps, but the overall level of adaptive convergence was low, demonstrating no shortage of alternative genomic solutions to cope with the challenges of the microclimate contrast. Mobile elements were a major source of genetic polymorphism and divergence, affecting all parts of the genome, including coding sequences of mating behavior-related genes.

## Significance

Adaptation to temperature and drought stress in *Drosophila* can be experimentally explored as a proxy model for adaptive trait complexes and genomic responses to climate variation. As a snapshot of synchronized adaptive events in a climate gradient, contemporary convergent evolution empowers the detection and understanding of adaptation from population genomic data and advance climate change assessment and forecasting. However, the effects of climate change on living organisms have been shown primarily on regional and global scales, confounding climate-related and climate-unrelated multivariate factors. This study leverages a unique microclimate contrast, known as Evolution Canyon, and a *Drosophila* model within it to provide a whole-genome perspective of adaptive evolution, convergence under thermal stress, and incipient speciation.

Climate variation and change are major abiotic stresses driving life's evolution (1–3). A classical observation in biogeography is the phenomenon of phenotypic convergence of life forms in areas of similar climate, a striking signature of evolutionary predictability (4, 5). The convergent evolution of similar attributes in response to shared selection pressures among disparate taxa is a testimony to the power of selection and its ability to repeatedly mold phenotypic variation. Processes contributing to phenotypic evolution other than selection, such as mutations and drift, are unlikely to generate the same evolutionary patterns time and again in correlation with environment (6).

Convergent phenotypes may originate through either divergent genetic solutions (7, 8) or the same pathways, genes, or even nucleotide positions (9, 10) in independent lineages. Convergence at the genetic level can in turn result from one of three processes: (*i*) evolution by mutations that occurred independently in different populations or species (parallel genetic evolution); (*ii*) evolution of an allele that was polymorphic in a shared ancestral population or species (transspecific polymorphism); and (*iii*) evolution of an allele that was introduced from one population into another by hybridization (introgression) (11, 12). Theoretical models predict that local standing genetic variation combined with spatial population structure limiting dispersal in an ecologically patchy environment largely facilitate rapid convergent evolution (13, 14). However, empirical tests of these predictions have become feasible only very recently due to the decreasing cost of population genomic sequencing.

Significant insights can be provided by local adaptations emerging across thermal gradients. These gradients are perhaps most dramatic in the Mediterranean region proper, where arid slope landforms produce local biodiversity refugia with microclimate contrasts of a magnitude equivalent to a multiyear temperature increase under rapid climatic change (15–17). One such site in particular, known as Evolution Canyon (Lower Nahal Oren, Mount Carmel, Israel), has long served as a natural system in which convergent local adaptations are observed in many taxa inhabiting this ecological microgradient (18–21). Greater solar radiation (up to 800% more) on the south-facing slope (SFS) is responsible for higher temperatures, drought, spatiotemporal heterogeneity, and fluctuation, as well as more xeric savannoid biota compared with the densely forested north-facing slope (NFS).

*Drosophila melanogaster* is an iconic example of a species with slope-specific adaptations in Evolution Canyon; SFS-derived flies outperform NFS-derived flies in basal and inducible thermotolerance after diverse heat shocks (22, 23), as well as in resistance to desiccation and starvation (22, 23). In addition, these two populations differ in phenotypic plasticity for wing morphology (24), oviposition site preferences (22), courtship song characteristics (25), and sexual and reproductive behavior (26) resulting in partial assortative mating within slopes (27). This differentiation is accompanied by divergence at the genome-wide and transcriptome-wide levels, including single nucleotide polymorphism (SNP) patterns within and outside coding sequences (27), repeat element profiles (28), as well as RNA editing (29). Remarkably, these genetic changes have accumulated despite the physical proximity and migration between slopes (30).

Evolution Canyon is inhabited by several other *Drosophila* species, including *Drosophila simulans*, a close relative of *D. melanogaster* with a similar but more recent history of out-of-Africa colonization of other continents (22). This co-occurrence of closely related species provides an attractive opportunity to investigate convergent evolution in response to the same microclimate contrast. Similar to *D. melanogaster*, *D. simulans* from SFS exhibited preference for higher oviposition temperature relative to conspecific females from NFS (22), but interslope divergence in this species has not been further surveyed. Here we present the analysis of *D. simulans* genomes and show that Evolution Canyon populations of this species are also characterized by interslope divergence with distinct adaptive signatures, even though the extent of evolutionary convergence between *D. melanogaster* and *D. simulans* at the genetic level is low.

## Results

### Genetic Polymorphism and Evolutionary Differentiation.

Genome pool-sequencing of 18 *D. simulans* isofemale lines resulted in 73× coverage (ranging from 62× to 82× per line) and an average mapping rate of 99.26% (*SI Appendix*, Table S1). We found a total of 4,564,564 SNP sites, including 9% synonymous substitutions and 4% were nonsynonymous substitutions (*SI Appendix*, Table S2). The principal component analysis (PCA) profile for all polymorphic sites showed more stratified diversification among NFS lines compared with SFS lines (*SI Appendix*, Fig. S1), strikingly similar to the pattern seen earlier in Evolution Canyon *D. melanogaster* (29). The two NFS lines clustering together with SFS could potentially be migrants from SFS, as some interslope migration of flies was in fact observed, and was found to be higher from SFS to NFS than in the opposite direction (30). Nevertheless, these two lines were kept for further analysis as NFS, consistent with the site of their collection.

**Fig. 1.**
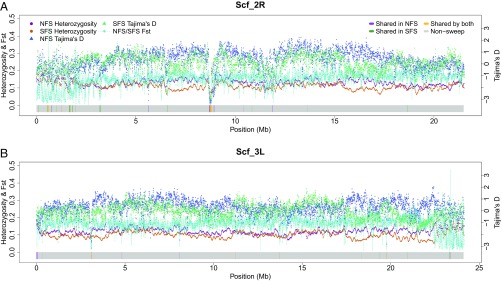
Heterozygosity, Tajima’s D, and F_ST_ values plotted against the putative selective sweep signatures (horizontal color blocks) along chromosomal arms 2R (*A*) and 3L (*B*) of *D. simulans*. The horizontal color blocks correspond to putative sweep regions shared by all lines (yellow), shared by all NFS lines (purple), shared by all SFS lines (green), and no sweep (gray).

The average interslope fixation index (F_ST_), a measure of population differentiation due to genetic structure, was 0.171, higher than the F_ST_ value recalculated for *D. melanogaster* (0.099) (29) using the same methods. Compared with NFS-, SFS-derived *D. simulans* were characterized by consistently lower levels of Tajima’s D, a statistic commonly used to summarize the site-frequency spectrum for SNP data, across all chromosomal arms except the X chromosome that had similar values to NFS, with the chromosomal arm 2L exhibiting the greatest difference (Fig. 1, Table 1, and *SI Appendix*, Fig. S2).

**Table 1.** Mean Tajima’s D, heterozygosity, and F_ST_ values per chromosomal arm

Chr	Tajima's D, NFS	Tajima's D, SFS	Heterozygosity, NFS	Heterozygosity, SFS	F_ST_
2L	0.3701	−0.0976	0.1344	0.1059	0.1589
2R	0.3501	0.0161	0.1303	0.1113	0.1532
3L	0.3040	−0.1040	0.1219	0.1010	0.1664
3R	0.3203	0.0270	0.1190	0.1052	0.1734
X	−0.7899	−0.9296	0.0965	0.0880	0.2006

Heterozygosity followed a similar pattern, with SFS producing consistently lower values, even though the differences were less pronounced (Fig. 1 and Table 1). Such differentiation patterns are expected to form in response to either pervasive selection or demographic effects of a bottleneck in SFS (or a combination of the two). If demography were the main driving force behind the patterns, one might expect the X chromosome, having three-quarters of the effective autosome population size, to be most affected and produce the largest interslope difference. Mean Tajima’s D values for X chromosome were negative and lower (or more negative) than for autosomes (Table 1); however, the mean difference in D between slopes was smaller for the X chromosome compared with autosomes. Interslope F_ST_ ranged between 0.153 for 2R and 0.201 for the X chromosome (Table 1).

We also sequenced 36 individual males (18 per slope) from a new set of isofemale lines established in 2018, with an average 59× coverage (36× to 77× per line) and average mapping rate 97.33% (*SI Appendix*, Table S1). A total of 3,881,816 SNP sites were found in this collection, including 11% synonymous substitutions and 4.6% nonsynonymous substitutions, with the overall pattern of heterozygosity being similar to that seen in the 2014 collection.

### Adaptive Divergence.

To characterize adaptive divergence, we looked into putative selective sweep regions prevalent among isofemale lines from one slope but absent or rare among those from the other slope, which can be measured as a “differential sweep score” for each gene. This score measures the relative abundance of putative selective sweep regions among NFS lines compared with SFS lines per gene (*SI Appendix*, *Materials and Methods*). Our differential sweep score was negatively correlated with mean interslope difference in Tajima’s D (Spearman’s *r* = −0.764; *P* < 2.2 × 10^−16^), as well as average heterozygosity (*r* = −0.764; *P* < 2.2 × 10^−16^). There were 59 genes in SFS-prevalent selective sweep regions and only 10 genes in NFS-prevalent selective sweep regions (differential sweep score ≥6) (*SI Appendix*, Table S3). These 69 genes were enriched for cation balance and membrane transport ontologies (*SI Appendix*, Table S4). We found a similar functional enrichment in *D. melanogaster* experimentally selected for increased desiccation resistance (31). The three genes with the highest differential sweep score (−8) were *pipsqueak* (*psq*), *CG32772*, and *proctolin receptor* (*proc*). The first two genes encode DNA-binding domains, whereas *proc* has an RNA-binding domain and an activity involved in a neuropeptide signaling pathway (32). We previously found two of the 69 *D. simulans* genes (3%), *G protein-coupled receptor kinase 2* and *NFAT nuclear factor*, within slope-specific selective sweep regions in *D. melanogaster* from Evolution Canyon as well (29). *Drosophila* NFAT, like mammalian NFAT5, regulates the electrochemical balance (33). A predominance of selective sweeps in SFS was also found in the 2018 collection, with a total of 350 (5.14 Mb) selective sweep regions in SFS, compared with 208 (3.39 Mb) in NFS (*SI Appendix*, Fig. S3 and Table S5). Interestingly, 10 mating behavior-related genes (*y*, *mbl*, *Gr66a*, *dsf*, *Hr39*, *Gr39a*, *ppk23*, *lov*, *Gr39b*, and *Adar*) were located in SFS selective sweep regions, while six such genes (*y*, *mbl*, *Gr66a*, *Gr39b*, *lov*, and *peb*) were found in NFS sweep regions. Genes within selective sweeps were enriched in muscle and nervous system development, insecticide response, and sensory perception (*SI Appendix*, Table S6). We found a similar pattern in *D. melanogaster*, with 371 (3.87 Mb) in NFS and 608 (7.22 Mb) in SFS (29).

**Fig. 2.**
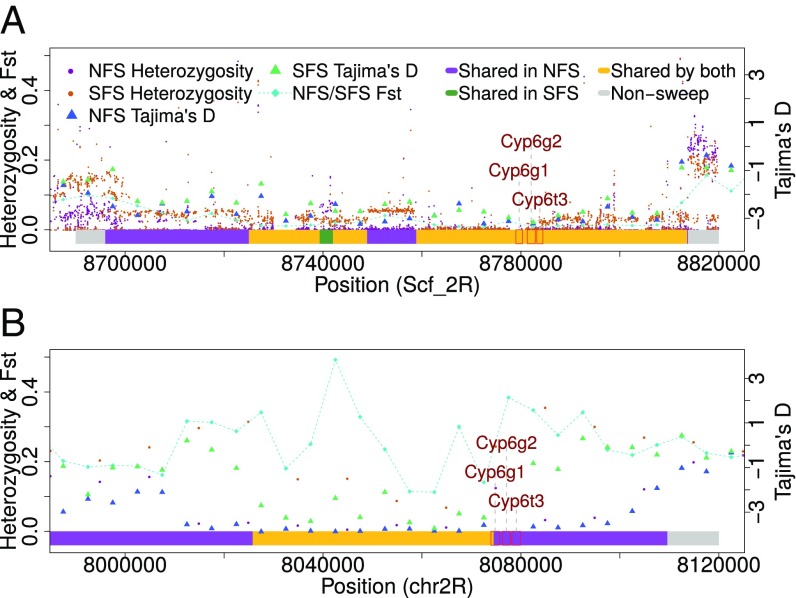
Steep decline of heterozygosity (based on 100-bp windows), Tajima’s D values on a chromosomal arm 2R region (8,700,000–8,820,000) in *D. simulans* (*A*), and the corresponding region (chr2R 8,000,000–8,120,000) in *D. melanogaster* (*B*). Colors used correspond to sweep regions as in Fig. 1.

We recorded a steep decline in Tajima’s D (down to an average of −1.691 in NFS and −1.338 in SFS) and heterozygosity (0.055 in NFS and 0.076 in SFS) on 2R between positions 8,700,000 and 8,820,000 in populations from both slopes and collections (Fig. 1 and *SI Appendix*, Table S5). The ∼120-kb interval contains 31 genes, but three of them—*Cyp6g1*, *Cyp6g2*, and *Cyp6t3*—had the most extreme Tajima’s D and heterozygosity values within a selective sweep region shared by NFS and SFS (Fig. 2*A*). All three genes encode cytochrome P450 enzymes, with at least two of them, *Cyp6g1* and *Cyp6g2*, responsible for acquired resistance to such insecticides as DDT, nitenpyram, dicyclanil, and diazinon (34). This selective sweep region is largely shared with *D. melanogaster* from both slopes (Fig. 2*B*) and appears to be a *D. simulans* genome feature with a worldwide distribution (35).

We found another steep decline in Tajima’s D (down to an average of −2.606 in NFS and −2.383 in SFS) and heterozygosity (0.037 in NFS and 0.074 in SFS) in 3L between positions 3,083,000 and 3,110,000 in populations from both slopes (Fig. 1). This region includes three genes—*Kap*, *Hsp83*, and *gry*—and is shared with *D. melanogaster* from both slopes as well (*SI Appendix*, Fig. S4). Of the three genes, only sequence variation in *Hsp83*, a major hub gene important for fecundity, longevity, and buffering of cryptic deleterious variation, was assayed in wild populations of *D. melanogaster* and was found to exert profound fitness effects (36).

### Convergent Evolution at the Genomic Level and Transspecies Polymorphism.

To investigate convergent evolution between *D. simulans* and *D. melanogaster* at the genome-wide level in a more systematic way, we estimated genewise Spearman’s rank correlations between species for differential sweep scores, as well as interslope F_ST_, mean interslope differences in Tajima’s D, and heterozygosity (Table 2). The near-zero correlations for all these parameters indicate very low levels of genetic convergence overall.

**Table 2.** Spearman rank correlations between scores of *D. simulans* and *D. melanogaster*

Category	*r*	*P* value
Sweep score	0.0326	0.0269
Difference in Tajima's D	0.0185	0.2091
Difference in heterozygosity	−0.0181	0.2194
F_ST_	−0.0414	0.0049

We reasoned that co-occurrence of shared (transspecies) polymorphisms between *D. simulans* and *D. melanogaster* from the same slope may provide finer-scale insights into convergent adaptive evolution of the system. There were 43,433 transspecies polymorphisms (<1% of all polymorphic SNPs in *D. simulans*) in these two species sampled in Evolution Canyon. Co-occurring major alleles were >2.5-fold enriched relative to nonshared alleles, but this enrichment was essentially independent of whether *D. simulans* and *D. melanogaster* originated from the same slope or the opposite slopes, across all genomic sites, CDS sites, and nonsynonymous sites (*SI Appendix*, Table S7). Spearman’s rank correlation between species with respect to interslope differences in shared allele frequencies was near zero and mostly nonsignificant (*SI Appendix*, Table S8). Only seven transspecies polymorphisms in nine genes—*CG7810*, *mus201*, *CG30466*, *CG8311*, *Elk*, *CG14492*, *nord*, *Ir60a*, and *CalpB*—were at the same time nonsynonymous, slope-divergent (i.e., alternative alleles predominant on opposing slopes), and shared by species within the slopes. For example, an SNP resulting in a change of serine to phenylalanine within *CG7810* (function unknown) occurred with frequency of 100% in NFS-derived *D. melanogaster* and 59% in NFS-derived *D. simulans*, while among SFS-derived flies, the allele frequency decreased to 35% and 30%, respectively (Fisher’s exact test, *P* < 0.0001).

### Repeatome Divergence.

**Fig. 3.**
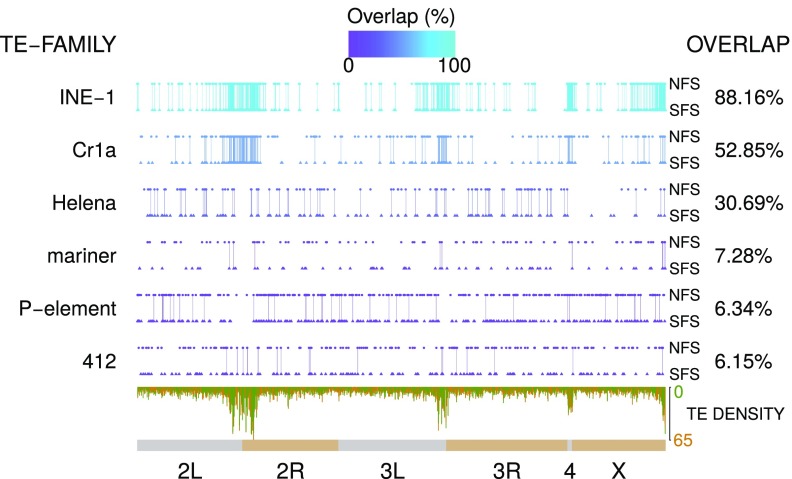
Examples of TE distributions along chromosomal arms: *INE-1*, *Cr1a*, *Helena*, *mariner*, *P-element*, and *412*. Each round dot represents one NFS insertion, and each triangle represents one SFS insertion. Connection lines between dots indicate sharing of same insertions. The color of the line for each TE family reflects the overlap percentage between NFS and SFS, from low (dark color, purple) to high (bright color, cyan). TE densities (green for NFS and orange for SFS) were calculated based on a 100-kb window.

Profiling of transposable elements (TEs) revealed a total of 9,036 TE insertions in NFS-derived and 9,182 in SFS-derived *D. simulans*, with chromosome 4 having the highest (24 per 100 kb) and chromosomal arm 3R the lowest (6.1 per 100 kb) TE density (Fig. 3 and *SI Appendix*, Table S9). A total of 4,207 TE insertions in NFS and 4,353 TE insertions in SFS were slope-specific (47%). Class I [long terminal repeat (LTR) and non-LTR] TEs accounted for 50% of all TEs, 16% less than in previously characterized Evolution Canyon *D. melanogaster* genomes (28). PCA profiles of TE insertions produced a differentiation pattern similar to that of SNPs, in which, unlike NFS lines, SFS lines formed a tight cluster (*SI Appendix*, Fig. S5). A retro-TE *roo*, with 610 copies in NFS and 554 copies in SFS, was the TE with the greatest copy number difference between slopes (Fisher’s exact test, *P* = 0.049) (*SI Appendix*, Table S10). The most divergent site due to TE polymorphism was an *INE-1* insertion within the 3′ UTR region of *sphinx2*, present in all nine SFS lines and in only one NFS line (*SI Appendix*, Table S11). Notably, *INE-1* was one of the least polymorphic TEs, as 908 out of 1,030 (88%) insertion sites were shared between slopes, suggesting that differentiation within *sphinx2* is more likely due to slope-divergent selective pressures than to recent *INE-1* transposition. The *sphinx2* gene is involved in innate immune responses and positive regulation of the Toll signaling pathway (37).

We then compared insertion polymorphisms among all TEs and found that *P*-element and retrotransposon *412* were the most variable TEs, with only 37 out of 584 *P*-element insertion sites (6%; Fisher’s exact test, *P* = 1.11 × 10^−66^) and 19 out of 309 *412*-element insertion sites (6%; *P* = 7.44 × 10^−36^) shared between slopes, followed by *mariner* (7%; *P* = 1.60 × 10^−16^) and *G*-element (7%; *P* = 5.25 × 10^−12^) (Fig. 3). As many as 519 TEs in NFS and 532 TEs in SFS disrupted coding sequences, including heat shock protein genes *Hsp23* (one SFS line) and *Hsp67Ba* (one NFS line and one SFS line), both disrupted by a *P*-element (*SI Appendix*, Table S12). Similar patterns were observed in the 2018 collection, with 76.28% *INE-1* insertions shared between the two slopes but only 7.41% *P-*element insertions found in both slopes. *GATE* was the most variable TE, with only 1.2% insertions shared between NFS and SFS (*SI Appendix*, Table S13). Interestingly, 41 and 48 TE insertions were found in the coding region of 29 and 33 mating behavior-related genes in NFS and SFS, respectively (*SI Appendix*, Table S14). Ten of these genes—*amn*, *btv*, *Dg*, *intr*, *lov*, *Pde1c*, *ple*, *pros*, *shep*, and *spin*—were found in both NFS and SFS, with putative TE insertions in CDS sites.

## Discussion

Like *D. melanogaster*, *D. simulans* is originally native to Africa but currently shows a widespread geographical distribution and has adapted to a wide variety of environments, including those in temperate climates. Despite relatively recent common ancestry (2–8 mya), phenotypic similarities, and largely shared habitats, *D. simulans* and *D. melanogaster* differ in a number of important ecophysiological traits (38). Notably, *D. simulans* is less resistant to temperatures outside the typical thermal range of 12–31 °C for these two species, as exemplified by a greater sensitivity to heat stress (39). This species is also characterized by lower tolerance of desiccation compared with *D. melanogaster* (reviewed in ref. 38). These ecophysiological differences may explain why *D. simulans*, being the species less resistant to climate-related stress factors, produced a stronger pattern of interslope divergence with distinct adaptive signatures along the microclimate contrast. *D. simulans* from SFS exhibited more extensive signatures of selective sweeps in general, and SFS-prevalent selective sweep regions were enriched in genes responsible for electrochemical gradient, a functional category previously associated with directional selection for increased desiccation resistance (31, 40).

However, it is interesting that *D. simulans* has been known to form no apparent clines for cold tolerance or heat shock in Australian populations of *D. simulans*, in contrast to the strongly clinal traits in Australian *D. melanogaster* (41). In addition, latitudinal variation at the genomic level has been found to be less pronounced in North American *D. simulans* than in North American *D. melanogaster* (42).

Regardless of large-scale biogeographic patterns, *D. simulans* and *D. melanogaster* clearly differ in their response to ecological challenges along the microclimate. Despite the presence of interslope divergence in both species, we found little evidence for parallel or convergent adaptations between *D. simulans* and *D. melanogaster* in Evolution Canyon at the genetic level. Except for a large selective sweep region in 2R shared across species and slopes, presumably associated with insecticide resistance (35), and another in 3L spanning *Hsp83*, there otherwise was little overlap between selective sweep positions. The paucity of convergent evolution seems to be at odds with intraspecies experimental evolution studies that typically reveal moderate to high levels of convergence, due mostly to standing genetic variation (e.g., ref. 43), as well as some natural systems, such as threespine stickleback (44). Interspecies convergence between two species is dependent on the frequency of similar or identical mutations occurring independently in both species, as well as shared alleles between them (12). Since new parallel mutations are rare, and the frequency of alleles shared by distinct species (represented by transspecies polymorphisms) is decreased relative to total levels of intraspecies polymorphism, low adaptive convergence between such divergent species as *D. simulans* and *D. melanogaster* is not unexpected.

This scarcity of adaptive convergence extends to transspecies polymorphisms, despite the excess of shared major alleles between *D. simulans* and *D. melanogaster*. The increased frequency of shared alleles among transspecies polymorphic loci was largely independent of the microclimate contrast, implying no or little convergence due to local adaptations. The overall excess of shared polymorphisms might have resulted from the constraints on the number of possible neutral allelic states, unless synonymous SNPs were in fourfold degenerate positions, or ancient transspecies polymorphism predating the divergence between the two species (45). The lack of interspecies correlation between interslope differences among shared nonsynonymous polymorphisms would also be expected if these sites were under long-term balancing selection unrelated to the microclimate contrast rather than convergent adaptive evolution, as exemplified by polymorphism in genes encoding secreted antimicrobial peptides in *D. melanogaster* and *D. simulans* (46).

Similar to sympatric *D. melanogaster* (28), nearly one-half of all mobile element insertions in *D. simulans* were slope-specific, providing an ample source of genetic variation for selection to act upon. The *P*-element was among the most polymorphic insertions, consistent with the dynamics of an element that invaded natural populations of *D. simulans* only recently, presumably through a single event of horizontal transfer from *D. melanogaster* (47). Incidentally, frequencies of the other two most polymorphic TEs, *412* and *mariner*, have been observed in *D. simulans* to correlate with temperature. The copy number of *412* increases from south to north following a temperature cline (48), whereas *mariner* activity tends to decrease in colder temperatures (49). While *412* copy numbers were almost identical between NFS (160) and SFS (168), *mariner* was indeed less abundant in NFS (69) than in SFS (93), even though the difference was not statistically significant (Fisher’s exact test, *P* = 0.08). We were particularly interested in insertion polymorphisms within promoter regions and coding sequences diverging between slopes, similar to that reported for *D. melanogaster*’s heat shock protein *Hsp70* (50, 51). However, apart from the *INE-1* insertion polymorphism in the 3′ UTR region of *sphinx2* that was highly slope-specific, we found little TE-caused divergence that would imply adaptive significance in the microclimate gradient. Overall TE differences likely reflect a combination of internal transposition dynamics (some of which can be environmentally sensitive), selection, and demography.

We previously reported 20 cognition-related and 17 sensory perception-related genes affected by TE inserts in *D. melanogaster* (28), including eight olfactory receptor and eight gustatory receptor genes, all critical for detecting food and avoiding toxicants, as well as for courtship and mating. Cognition, sensory perception of chemical stimuli, and olfaction were among the most significantly overrepresented GO terms among genes with TE-disrupted coding sequences in *D. melanogaster* (28). We and others have observed various degrees of partial mating isolation between NFS- and SFS-derived *D. melanogaster* over many years of fly collections in Evolution Canyon (26, 27, 52) (but see ref. 53 for an exception). We did not investigate mating discrimination and courtship behavior in *D. simulans* from the canyon but note that, similar to *D. melanogaster*, there is ample polymorphism in mating behavior genes due to TE insertions within their coding sequences, some of which slope-specific.

## Conclusions and Future Directions

We conclude that despite being a species with a more recent out-of-Africa colonization history than *D. melanogaster*, *D. simulans* is characterized by very distinct interslope genomic differentiation, with signatures of adaptive evolution prevalent among flies from the temperature-stressful SFS. To investigate this model further, it will be important to assess interslope phenotypic differentiation in *D. simulans*, including stress-related performance and mating preferences, and to profile associated transcriptomes and RNA-editing patterns (29).

## Materials and Methods

### Fly Collections.

*D. simulans* females inseminated in nature were collected on the opposite slopes of Evolution Canyon (Nahal Oren, Mount Carmel, Israel) on October 26, 2014. The descendants were kept as isofemale lines on instant *Drosophila* medium (Carolina Biological Supply) in 0.5-pint bottles at a temperature of 24 ± 1 °C and on a 12:12 light/dark cycle. For comparison, *D. melanogaster* from the same locations, collected at the same time, and maintained under the same conditions along with *D. simulans* were used, as characterized by Yablonovitch et al. (29). An additional set of *D. simulans* collected at the same sites in May 2018 was later added. (No *D. melanogaster* were found at that time.)

### Sampling and DNA Extractions.

We used 9 SFS lines and 9 NFS isofemale lines from the 2014 collection and 18 SFS lines and 18 NFS isofemale lines from the 2018 collection. DNA was extracted from 20 females and pooled per line from the 2014 collection using a standard Qiagen protocol (Gentra Purgene Tissue Kit). For the 2018 collection, DNA was extracted from a single male per line using the same protocol. TruSeq DNA libraries were prepared and sequenced on the HiSeq platform following Illumina’s protocols, and 2 × 150-bp paired-end reads were generated (*SI Appendix*, Table S1).

### Mapping Reads and Data Processing.

The *D. simulans* genome (dsim_r2.02) and corresponding annotations from FlyBase (http://flybase.org/) served as a reference for mapping. Raw reads were quality controlled and filtered with FastqMcf (54). The remaining reads were mapped to the reference using BWA (55) with default parameters. GATK (56) with default parameters (except for using “–sample_ploidy” for pooled data and setting –heterozygosity to 0.01) was used for genotyping each sample. Genotypes with more than two alleles were discarded. Only sites with genotyping quality >30, a minimum depth of 10, and a maximum depth of 250 were used in the analysis.

### Estimates of F_st_, θ, Tajima’s D, and Heterozygosity.

Samtools (57) was used to generate the pileup file (−Q 20). SNPs within 10 bp of indels were discarded. An F_ST_ value for each SNP was generated using PoPoolation2 (58), whereas Watterson’s θ and Tajima’s D were calculated PoPoolation (59). Tajima’s D, heterozygosity, and the average F_ST_ value were calculated for a window size of 5 kb unless stated otherwise, as well as for each gene.

### Selective Sweeps Detection and Differential Sweep Score.

Pool-hmm (60) was used for finding selective sweep signatures from pool-seq data in each NFS and SFS line. This hidden Markov model (HMM)-based method estimates the allele frequency spectrum and detects a selective sweep if the hidden state “Selection,” corresponding to swept or near-swept positions, is inferred for a window of sites. This HMM approach has similar power to detect selective sweeps but is more robust to demographic events (61) than the original method of Kim and Stephan (62), which uses the full site frequency spectrum information to test the significance of variation reduction and frequency spectrum skew due to hitchhiking event around the selected site (62). The input pileup files to the Pool-hmm pipeline were generated using samtools (57) after reads were mapped to the reference genome. The parameters used in Pool-hmm pipeline were “-n 40 -c 5 -C 250 -q 20 -p -k 0.0000000001” (number of haplotypes, 40; minimum coverage, 5; maximum coverage, 250; per site transition probability, 0.0000000001, as suggested in ref. 60), while “–theta” was set to be the θ estimated individually for each line and sweep regions were reported. For each gene, we counted the number of NFS line (*N*) in which this gene was reported within the putative sweep region of this line, as well as such number of SFS line (*S*). The differential sweep score for each gene was calculated as the difference of the two numbers (*N* − *S*). In this study, the differential sweep scores range from −9 to 9. The corresponding differences of Tajima’s D values and heterozygosity were generated in the same manner.

### Identification of TE Insertions.

TE insertions were identified with PoPoolation TE (63), and TE sequences were downloaded from FlyBase (transposon sequence v9.42; http://flybase.org/). To minimize the effect of different sequencing depths to TE identification, a randomly selected subset of mapped reads from each sample was used in TE identification, each containing the same number of reads. Interslope divergence scores for TEs were calculated in the same manner as the sweep scores.

### Data Availability.

All sequencing data have been deposited to the NCBI Sequence Read Archive (SRA) under accession no. SRP132777.

## Supplementary Material

Supplementary File
