# Supplementary material for: Genomic divergence and adaptive convergence in Drosophila simulans from Evolution Canyon, Israel
Source: Proc Natl Acad Sci U S A. 2019 May 24;116(24):11839–44. doi: 10.1073/pnas.1720938116 (PMC6576144; doi:10.1073/pnas.1720938116)
Supplement: Supplementary File [file pnas.1720938116.sapp.pdf]

SI Appendix

Tables

Table S1. Summary of genome sequencing.

Table S2. Distribution of SNPs along genomic regions.

Table S3. List of genes with sweep score above or equal to 6 (|score| >=6).

Table S4. GO term enrichments of genes with sweep score above or equal to 6 (|score| >=6).

Table S5. Selective sweeps identified in the 2018 collection.

Table S6. GO enrichment analysis of genes within selective sweep regions in the 2018 data.

Table S7. Counts of co-occurring major alleles between *D. simulans* and *D. melanogaster* across all trans-species polymorphic sites.

Table S8. Correlation of changes of frequency of two slopes for trans-species polymorphic sites between *D. simulans* and *D. melanogaster*.

Table S9. Distribution of TEs across chromosomal arms.

Table S10. Number of TEs in each family for NFS and SFS.

Table S11. List of TE insertions with extreme TE scores (|score|>=6).

Table S12. List of insertions within the CDS regions per TE families *412*, *P-element*, *mariner* and *G-element*.

Table S13. List of TEs found in 2018 data.

Table S14. List of TE insertions in CDS regions of mating behavior-related genes from 2018 data.

www.pnas.org/cgi/doi/10.1073/pnas.1720938116

Figures

Fig. S1. PCA plot of SNPs from 9 NFS (red) lines and 9 SFS (turquoise) *D. simulans* isofemale lines.

Fig. S2. Heterozygosity, Tajima's D and F_{ST} values plotted against the putative selective sweep signatures (horizontal color blocks) along *D. simulans* chromosomal arms 2L (A), 2R (B), 3L (C), 3R (D), and X (E). Horizontal color blocks correspond to putative sweep regions: shared by all lines (yellow), shared by all NFS lines (purple), shared by all SFS lines (green), and no sweep (grey).

Fig. S3. Length of selective sweeps found in NFS and SFS from the 2018 collection.

Fig. S4. Steep decline of heterozygosity (based on 100 bp window) and Tajima's D values on a chromosomal arm 3L region (3,083,000 to 3,110,000) in *D. simulans.*

Fig. S5. PCA plot profiling TEs from 9 NFS (red) lines and 9 SFS (turquoise) lines. Ellipse indicates the normal data with probability of 0.9.

Table S1. Summary of genome sequencing.

	SampleID	Group	NumOfReads	TotalBase	Q20(%)	Q30(%)	Depth(X)*	GCContent (%)	MappingRate (%)
2014 collection	NFS51	NFS	56,118,794	8,434,266,307	97.74%	93.58%	65.90	44.48%	98.94%
	NFS55	NFS	70,115,702	10,549,087,182	97.63%	93.23%	82.42	44.85%	99.41%
	NFS56	NFS	53,799,628	8,092,338,010	97.65%	93.36%	63.23	44.01%	99.12%
	NFS58	NFS	65,443,110	9,852,479,679	97.54%	93.03%	76.98	44.80%	99.64%
	NFS61	NFS	63,243,952	9,511,914,079	97.68%	93.41%	74.32	44.35%	99.59%
	NFS63	NFS	63,106,398	9,494,179,817	97.63%	93.25%	74.18	44.66%	99.08%
	NFS64	NFS	63,282,708	9,523,660,630	97.63%	93.33%	74.41	45.04%	99.71%
	NFS65	NFS	61,214,512	9,206,604,275	97.49%	92.99%	71.94	43.43%	99.17%
	NFS68	NFS	59,671,864	8,975,846,887	97.72%	93.52%	70.13	43.98%	99.16%
	SFS16	SFS	53,407,886	8,031,412,535	97.66%	93.42%	62.75	43.34%	99.13%
	SFS36	SFS	69,775,044	10,497,149,269	97.59%	93.22%	82.02	43.60%	99.16%
	SFS38	SFS	61,534,212	9,258,531,778	97.72%	93.53%	72.34	43.74%	99.16%
	SFS39	SFS	56,175,690	8,458,513,407	97.57%	93.13%	66.09	43.76%	99.28%
	SFS59	SFS	69,077,262	10,396,771,003	97.78%	93.65%	81.23	44.90%	99.48%
	SFS62	SFS	68,033,538	10,240,440,794	97.70%	93.44%	80.01	44.32%	99.29%
	SFS65	SFS	63,718,718	9,586,998,186	97.74%	93.59%	74.91	43.62%	98.66%
	SFS69	SFS	61,327,252	9,214,891,757	97.67%	93.37%	72.00	44.22%	99.21%
	SFS70	SFS	69,696,220	10,481,164,120	97.74%	93.55%	81.89	44.86%	99.52%
2018 collection	NFS10	NFS	47,499,022	7,039,549,461	98.35%	95.12%	55.00	43.86%	98.75%
	NFS11	NFS	59,235,800	8,715,567,803	98.38%	95.20%	68.10	44.37%	97.68%
	NFS12	NFS	56,856,396	8,448,801,503	98.31%	95.02%	66.01	44.20%	97.41%
	NFS13	NFS	43,471,762	6,453,829,250	98.37%	95.15%	50.43	44.25%	98.70%
	NFS15	NFS	60,149,998	8,861,468,439	98.33%	95.07%	69.24	44.68%	96.87%
	NFS16	NFS	66,206,930	9,725,636,680	98.40%	95.24%	75.99	44.22%	98.16%
	NFS18	NFS	43,701,310	6,517,561,428	98.25%	94.95%	50.92	42.66%	98.74%
	NFS19	NFS	59,846,814	8,834,539,436	98.38%	95.19%	69.03	44.42%	96.17%
	NFS1	NFS	67,000,136	9,829,223,359	98.29%	94.96%	76.80	44.18%	98.91%
	NFS20	NFS	65,668,368	9,664,406,049	98.39%	95.24%	75.51	44.26%	97.77%
	NFS2	NFS	60,025,290	8,878,201,511	98.25%	94.83%	69.37	44.54%	97.73%
	NFS3	NFS	47,245,174	7,000,233,217	98.17%	94.65%	54.70	44.28%	92.69%
	NFS4	NFS	54,764,018	8,107,449,765	98.17%	94.71%	63.35	43.10%	98.54%
	NFS5	NFS	45,261,688	6,710,892,269	98.20%	94.75%	52.44	43.20%	95.98%
	NFS6	NFS	50,382,790	7,408,215,223	98.26%	94.83%	57.88	45.84%	92.90%
	NFS7	NFS	31,112,946	4,602,811,730	98.40%	95.24%	35.96	44.24%	98.84%
	NFS8	NFS	58,419,294	8,555,846,152	98.37%	95.09%	66.85	47.13%	87.58%
	NFS9	NFS	55,607,990	8,238,755,461	98.33%	95.10%	64.37	43.12%	97.19%
	SFS11	SFS	40,938,068	6,051,148,710	98.04%	94.32%	47.28	42.25%	97.76%
	SFS12	SFS	55,438,798	8,212,872,112	98.08%	94.42%	64.17	43.58%	97.82%
	SFS14	SFS	51,033,548	7,559,124,906	98.09%	94.40%	59.06	43.98%	98.97%
	SFS15	SFS	41,286,712	6,128,362,516	98.08%	94.55%	47.88	42.53%	98.29%
	SFS16	SFS	51,416,074	7,600,555,837	98.27%	94.90%	59.39	43.59%	97.36%
	SFS17	SFS	59,246,346	8,753,511,422	98.27%	94.91%	68.39	44.17%	98.77%
	SFS18	SFS	44,879,274	6,635,193,978	98.18%	94.74%	51.84	42.19%	97.08%
	SFS19	SFS	48,646,212	7,148,647,891	98.28%	94.93%	55.86	44.09%	98.84%
	SFS1	SFS	48,641,068	7,196,241,115	98.05%	94.36%	56.23	43.14%	98.33%
	SFS20	SFS	42,228,122	6,268,577,238	98.21%	94.76%	48.98	44.21%	95.55%
	SFS2	SFS	57,808,982	8,488,050,591	98.15%	94.54%	66.32	44.43%	98.42%

SFS3	SFS	41,501,476	6,136,133,019	98.08%	94.43%	47.94	42.12%	98.48%
SFS4	SFS	49,206,426	7,229,293,368	98.05%	94.37%	56.49	42.16%	99.02%
SFS5	SFS	50,983,610	7,484,057,600	98.06%	94.37%	58.48	43.36%	98.19%
SFS6	SFS	41,009,852	6,039,362,168	98.10%	94.43%	47.19	43.72%	98.11%
SFS7	SFS	52,464,386	7,653,486,425	98.08%	94.46%	59.80	42.47%	98.71%
SFS8	SFS	39,788,856	5,843,775,265	98.09%	94.42%	45.66	43.90%	96.59%
SFS9	SFS	52,708,412	7,793,851,473	98.11%	94.45%	60.90	44.25%	96.93%

*Depth was calcuated with dividing the total bases by the genome reference size.

Table S2. Distribution of SNPs along genomic regions.

Region	Numer of sites	Percentage	Sequencing coverage	Density of SNP (per Kb)
3'-UTR	203,963	4.47%	7,217,040	28.26
5'-UTR	136,005	2.98%	4,308,515	31.57
CDS (Synonymous)	417,444	9.15%	21,918,482	
CDS (Non-Synonymous)	184,531	4.04%		27.46
Intergenic	1,555,308	34.07%	34,234,461	45.43
Intron	2,009,884	44.03%	46,762,163	42.98
Promoter*	57,429	1.26%	1,019,289	56.34
Total	4,564,564	100.00%	115,459,950	39.53

*: Promoter is defined as the 100bp region upstream of 5'-UTR

Table S3. List of genes with sweep score above or equal to 6 (|score| >=6; ^: shared with NFS in D. mel; *: shared with SFS in D. mel.)

GENE ID	GENE SYMBOL	SWEEP SCORE	DIFFERENCE IN D	DIFFERENCE IN HET	FST
FBgn0182509	psq	-8	1.51529	0.0882556	0.171418056
FBgn0188302	CG32772	-8	3.12791	0.0993222	0.137536111
FBgn0196066	Proc	-8	2.69784	0.130344	0.222898611
FBgn0187897	CG4041	-7	3.12825	0.0987667	0.165001389
FBgn0187918	CG11444	-7	1.57228	0.0754222	0.250275
FBgn0188285	CG3527	-7	2.07527	0.157267	0.239356944
FBgn0188301	CG6927	-7	3.43591	0.124656	0.149441667
FBgn0188303	CG6903	-7	2.74587	0.108344	0.187055556
FBgn0192995	CG18673	-7	3.5123	0.112822	0.169847222
FBgn0192996	ppk24	-7	3.09732	0.115967	0.220068056
FBgn0193715	CG44153	-7	1.44085	0.0684667	0.169134722
FBgn0268973	CG34188	-7	1.86606	0.111378	0.192538889
FBgn0269615	Muc68E	7	-2.36584	-0.0971222	0.124719444
FBgn0182356	-	-6	1.39105	0.0958111	0.095769444
FBgn0182511	CG11883	-6	1.84273	0.109022	0.183991667
FBgn0182871	CG43729	-6	1.05481	0.0846556	0.167705556
FBgn0183286	Cib2	-6	1.62921	0.115844	0.234995833
FBgn0184041	CG3797	-6	2.89912	0.112744	0.206677778
FBgn0184555	JIL	-6	1.28848	0.0677444	0.179931944
FBgn0185980	CG14141	-6	2.44418	0.0835889	0.187805556
FBgn0186640	CG7611	-6	1.44924	0.0758667	0.144552778
FBgn0186736	nrm	-6	2.86578	0.101467	0.151826389
FBgn0187896	CG44774	-6	0.665975	0.0459111	0.195566667
FBgn0187919	CG11436	-6	2.91357	0.153167	0.241525
FBgn0188094	CG1750	-6	2.26104	0.0893667	0.210029167
FBgn0188172	mesh	-6	1.88043	0.0606111	0.177023611
FBgn0188182	stops	-6	2.08004	0.0750556	0.167354167
FBgn0190641	Dad	-6	0.831519	0.0438889	0.163298611
FBgn0190865	H	-6	2.53785	0.0980444	0.107641667
FBgn0191064	CG11373	-6	0.9418	0.0815556	0.241798611
FBgn0191180	Vps24	-6	2.5585	0.190344	0.252527778
FBgn0191205	MP1	-6	2.05952	0.0811778	0.1625875
FBgn0191206	Suv3	-6	1.21634	0.142344	0.232534722
FBgn0191473	SIFaR	-6	2.18211	0.112333	0.171451389
FBgn0191529	Pi3K92E	-6	2.06422	0.121944	0.169694444
FBgn0192991	mRpL32	-6	1.91574	0.0509889	0.137536111
FBgn0192992	spn	-6	2.0472	0.0748667	0.163456944
FBgn0192993	CG3669	-6	2.58479	0.0942889	0.181472222
FBgn0192994	CG18672	-6	1.8948	0.0969222	0.214580556
FBgn0193003	Gprk2*	-6	1.22824	0.0426556	0.181855556
FBgn0193939	CG17376	-6	0.942361	0.0444889	0.1299375
FBgn0193941	nrv2	-6	1.07096	0.0654333	0.153565278
FBgn0193942	nrv1	-6	0.819132	0.0737889	0.127031944
FBgn0193953	nAChRalpha4	-6	0.911224	0.0514111	0.119020833
FBgn0194704	Col4a1	-6	1.28325	0.0919	0.156498611
FBgn0195034	CG34109	-6	1.79242	0.0814556	0.1717625

FBgn0195037	CG4839	-6	2.26708	0.0958111	0.173251389
FBgn0195926	CG5062	-6	2.16116	0.0745667	0.223197222
FBgn0268355	-	-6	1.87421	0.0747	0.138263889
FBgn0268802	-	-6	1.76804	0.0994778	0.248
FBgn0269036	CG3669	-6	2.7221	0.0938111	0.198840278
FBgn0269073	CG43245	-6	1.31037	0.0612	0.134133333
FBgn0269508	-	-6	0.345313	0.0265778	0.087911111
FBgn0269641	CG34221	-6	2.00405	0.0852444	0.156936111
FBgn0269657	-	-6	0.523523	0.0890667	0.205465278
FBgn0270181	-	-6	1.36871	0.0835778	0.118013889
FBgn0270450	-	-6	1.57812	0.0919222	0.162270833
FBgn0264036	mir-2577	-6	-0.00665802	0.131433	0.126290278
FBgn0183687	nvd	-6	0.85393	0.0949889	0.094868056
FBgn0183232	Obp56i	-6	2.26711	0.0956556	0.222490278
FBgn0184484	CG42397	6	-0.774614	-0.0698333	0.210952778
FBgn0187532	CG12177	6	-2.07187	-0.105433	0.227447222
FBgn0188706	NFAT	6	-1.95933	-0.077	0.2302125
FBgn0188708	CG11162	6	-1.95222	-0.128122	0.263523611
FBgn0193111	Hr38	6	-1.24827	-0.0384778	0.146152778
FBgn0268212	NFAT	6	0.114809	-0.0329667	0.170655556
FBgn0268566	NFAT	6	0.045253	-0.0241	0.135833333
FBgn0269939	Yp3	6	-1.32952	-0.0334	0.262402778
FBgn0270801	NFAT*	6	-0.518105	-0.0539556	0.184083333

Table S4. GO term enrichments of genes with sweep score above or equal to 6 (|score| >=6).

Category	Term	Count	PValue	Genes	FDR (%)
GOTERM_BP_DIRECT	GO:0098655~cation transmembrane transport	3	4.80E-04	NACHRALPH	0.596863202
GOTERM_MF_DIRECT	GO:0004089~carbonate dehydratase activity	3	0.00189	CG18672, C(	1.947922491
GOTERM_BP_DIRECT	GO:0006730~one-carbon metabolic process	3	0.00247	CG18672, C(	3.034613163
KEGG_PATHWAY	dme00910:Nitrogen metabolism	3	0.00351	CG18672, C(	2.871637887
GOTERM_BP_DIRECT	GO:0010248~establishment or maintenance of tra	2	0.00744	NRV1, NRV2	8.889891357
GOTERM_BP_DIRECT	GO:0090662~ATP hydrolysis coupled transmemb	2	0.00744	NRV1, NRV2	8.889891357
GOTERM_BP_DIRECT	GO:0006814~sodium ion transport	3	0.01778	NRV1, PPK2	20.03382864
GOTERM_CC_DIRECT	GO:0005890~sodium:potassium-exchanging ATP	2	0.0276	NRV1, NRV2	23.03987505
GOTERM_MF_DIRECT	GO:0008324~cation transmembrane transporter a	2	0.03229	NRV1, NRV2	28.94091172
GOTERM_MF_DIRECT	GO:0005391~sodium:potassium-exchanging ATP	2	0.03229	NRV1, NRV2	28.94091172
GOTERM_BP_DIRECT	GO:0035264~multicellular organism growth	2	0.04028	PI3K92E, NV	40.08647573
GOTERM_BP_DIRECT	GO:0036335~intestinal stem cell homeostasis	2	0.07552	PI3K92E, H	62.40925037
GOTERM_CC_DIRECT	GO:0005886~plasma membrane	7	0.08935	PI3K92E, CG	58.34293027
GOTERM_BP_DIRECT	GO:0002168~instar larval development	2	0.09605	PI3K92E, NV	71.58642127
GOTERM_BP_DIRECT	GO:0006813~potassium ion transport	2	0.09605	NRV1, NRV2	71.58642127

Table S5. Selective sweeps identified in the 2018 collection.

Slope	Chromosome	Start	End
NFS	Scf_2L	3242	9732
NFS	Scf_2L	11746	23490
NFS	Scf_2L	80563	92164
NFS	Scf_2L	196702	210684
NFS	Scf_2L	294099	305470
NFS	Scf_2L	1450693	1459268
NFS	Scf_2L	1985723	1996740
NFS	Scf_2L	3932523	3938646
NFS	Scf_2L	4992281	4998335
NFS	Scf_2L	19671936	19676153
NFS	Scf_2L	20528559	20540544
NFS	Scf_2L	20553949	20571243
NFS	Scf_2L	20717237	20724938
NFS	Scf_2L	20726919	20792478
NFS	Scf_2L	20876480	20887230
NFS	Scf_2L	20908248	20924948
NFS	Scf_2L	20943118	20959829
NFS	Scf_2L	20996001	21002661
NFS	Scf_2L	21041126	21061553
NFS	Scf_2L	21201785	21228390
NFS	Scf_2L	21333346	21346258
NFS	Scf_2L	21506479	21512621
NFS	Scf_2L	21543516	21567529
NFS	Scf_2L	21571393	21581584
NFS	Scf_2L	21642489	21649054
NFS	Scf_2L	21686339	21700898
NFS	Scf_2L	21712107	21722483
NFS	Scf_2L	21752248	21765018
NFS	Scf_2L	21786003	21791528
NFS	Scf_2L	21959981	21967565
NFS	Scf_2L	22028953	22032938
NFS	Scf_2L	22049913	22055721
NFS	Scf_2L	22058501	22066105
NFS	Scf_2L	22190509	22201613
NFS	Scf_2L	22218892	22264041
NFS	Scf_2L	22385036	22393620
NFS	Scf_2L	22504057	22511511
NFS	Scf_2L	22755977	22759283
NFS	Scf_2L	22841824	22844662
NFS	Scf_2L	22960152	22963644
NFS	Scf_2L	22992596	23007213
NFS	Scf_2L	23114472	23127023
NFS	Scf_2L	23162297	23179752
NFS	Scf_2L	23188654	23191488
NFS	Scf_2L	23265795	23276881
NFS	Scf_2L	23308755	23329055
NFS	Scf_2L	23361824	23365714
NFS	Scf_2L	23376695	23381176
NFS	Scf_2L	23406907	23417491

NFS	Scf_2L	23422826	23436626
NFS	Scf_2L	23455324	23460094
NFS	Scf_2L	23524571	23533287
NFS	Scf_2R	10783	15700
NFS	Scf_2R	287810	296623
NFS	Scf_2R	372327	378324
NFS	Scf_2R	393856	396724
NFS	Scf_2R	567775	587526
NFS	Scf_2R	655823	661431
NFS	Scf_2R	677819	687967
NFS	Scf_2R	1064964	1075854
NFS	Scf_2R	1108126	1143191
NFS	Scf_2R	1188147	1198965
NFS	Scf_2R	1211773	1224765
NFS	Scf_2R	1272423	1283984
NFS	Scf_2R	1367276	1377378
NFS	Scf_2R	1566279	1590185
NFS	Scf_2R	1615367	1639378
NFS	Scf_2R	1647369	1670791
NFS	Scf_2R	1683904	1691931
NFS	Scf_2R	1742555	1753848
NFS	Scf_2R	1803998	1814365
NFS	Scf_2R	3015099	3021020
NFS	Scf_2R	3232123	3240022
NFS	Scf_2R	4515405	4522896
NFS	Scf_2R	4826364	4834135
NFS	Scf_2R	5638227	5659603
NFS	Scf_2R	6536279	6563416
NFS	Scf_2R	8674212	8688939
NFS	Scf_2R	8693213	8812215
NFS	Scf_2R	8862759	8876569
NFS	Scf_2R	8881056	8897072
NFS	Scf_2R	8911440	8936031
NFS	Scf_2R	8956445	8961827
NFS	Scf_2R	8985879	8994161
NFS	Scf_2R	10387360	10392693
NFS	Scf_2R	10395142	10405039
NFS	Scf_2R	10842716	10848010
NFS	Scf_2R	11229903	11249009
NFS	Scf_2R	11283634	11288607
NFS	Scf_2R	11681794	11689686
NFS	Scf_2R	11794192	11801344
NFS	Scf_2R	11804127	11847320
NFS	Scf_2R	11853244	11857877
NFS	Scf_2R	11865276	11880180
NFS	Scf_2R	12112824	12119526
NFS	Scf_2R	13894978	13921519
NFS	Scf_2R	18060480	18070515
NFS	Scf_2R	18898285	18905711
NFS	Scf_2R	21392926	21413189
NFS	Scf_2R	21529878	21544591
NFS	Scf_3L	40285	56313

NFS	Scf_3L	96082	103805
NFS	Scf_3L	112420	122763
NFS	Scf_3L	314753	320862
NFS	Scf_3L	2648442	2657035
NFS	Scf_3L	3086823	3110760
NFS	Scf_3L	4682349	4688546
NFS	Scf_3L	5925815	5933245
NFS	Scf_3L	6048592	6053725
NFS	Scf_3L	8075615	8111130
NFS	Scf_3L	9353116	9359387
NFS	Scf_3L	11212457	11229865
NFS	Scf_3L	11233024	11239649
NFS	Scf_3L	12033824	12054404
NFS	Scf_3L	13751356	13762006
NFS	Scf_3L	17377609	17384914
NFS	Scf_3L	18267519	18273898
NFS	Scf_3L	18362374	18370571
NFS	Scf_3L	19765979	19783125
NFS	Scf_3L	20927762	20939657
NFS	Scf_3L	21382286	21387304
NFS	Scf_3L	22598255	22604194
NFS	Scf_3L	22715149	22750190
NFS	Scf_3L	22751836	22753615
NFS	Scf_3L	23083974	23090422
NFS	Scf_3L	23157454	23163675
NFS	Scf_3L	23372984	23379875
NFS	Scf_3L	23662987	23672923
NFS	Scf_3L	23926950	23930437
NFS	Scf_3R	101497	115247
NFS	Scf_3R	215899	235335
NFS	Scf_3R	1484063	1514529
NFS	Scf_3R	3479478	3509791
NFS	Scf_3R	5666833	5676205
NFS	Scf_3R	5702345	5715662
NFS	Scf_3R	6026526	6034785
NFS	Scf_3R	11758447	11779801
NFS	Scf_3R	11810794	11839844
NFS	Scf_3R	11920210	11945176
NFS	Scf_3R	12004953	12028845
NFS	Scf_3R	12055626	12097879
NFS	Scf_3R	14732130	14747733
NFS	Scf_3R	19529061	19538529
NFS	Scf_3R	19553307	19560121
NFS	Scf_3R	20774151	20786205
NFS	Scf_3R	20802438	20808417
NFS	Scf_3R	23075116	23085117
NFS	Scf_3R	23091520	23099111
NFS	Scf_3R	25580107	25600788
NFS	Scf_3R	27021589	27044960
NFS	Scf_3R	27048936	27063161
NFS	Scf_3R	27120266	27151410
NFS	Scf_X	10173	97988

NFS	Scf_X	98447	221937
NFS	Scf_X	250505	319588
NFS	Scf_X	439077	482960
NFS	Scf_X	1104735	1117072
NFS	Scf_X	1419461	1448097
NFS	Scf_X	1624034	1651417
NFS	Scf_X	1657353	1683587
NFS	Scf_X	1770584	1786246
NFS	Scf_X	2029070	2039182
NFS	Scf_X	2590525	2599089
NFS	Scf_X	3375582	3397386
NFS	Scf_X	3654448	3661280
NFS	Scf_X	3860345	3877951
NFS	Scf_X	4204376	4213406
NFS	Scf_X	4219429	4227709
NFS	Scf_X	5470978	5481099
NFS	Scf_X	5493315	5523627
NFS	Scf_X	5741869	5750443
NFS	Scf_X	6056285	6064492
NFS	Scf_X	6126408	6132005
NFS	Scf_X	6238810	6256081
NFS	Scf_X	6346425	6356190
NFS	Scf_X	6526093	6542068
NFS	Scf_X	7369601	7389991
NFS	Scf_X	8352052	8360198
NFS	Scf_X	8594554	8612328
NFS	Scf_X	8670342	8690256
NFS	Scf_X	8777002	8824785
NFS	Scf_X	8828622	8862918
NFS	Scf_X	8961909	9064655
NFS	Scf_X	9110760	9183133
NFS	Scf_X	10130951	10139263
NFS	Scf_X	10489866	10499419
NFS	Scf_X	12690046	12707690
NFS	Scf_X	12924734	12943688
NFS	Scf_X	13167851	13178343
NFS	Scf_X	13430195	13459196
NFS	Scf_X	13519037	13538362
NFS	Scf_X	14080106	14086650
NFS	Scf_X	14307083	14326561
NFS	Scf_X	14345833	14358573
NFS	Scf_X	14445331	14453625
NFS	Scf_X	15942666	15948743
NFS	Scf_X	15966448	15996299
NFS	Scf_X	16012782	16025480
NFS	Scf_X	16213039	16235847
NFS	Scf_X	16242587	16265489
NFS	Scf_X	16277322	16298271
NFS	Scf_X	17326085	17333799
NFS	Scf_X	19219799	19235253
NFS	Scf_X	19255682	19262792
NFS	Scf_X	19366461	19379407

NFS	Scf_X	19761198	19767977
NFS	Scf_X	20310076	20316560
NFS	Scf_X	20822983	20829645
SFS	Scf_2L	3264	29288
SFS	Scf_2L	66775	121560
SFS	Scf_2L	196950	210696
SFS	Scf_2L	294128	305463
SFS	Scf_2L	1452357	1461914
SFS	Scf_2L	3929084	3939536
SFS	Scf_2L	3988328	3998037
SFS	Scf_2L	4643484	4649406
SFS	Scf_2L	5634930	5645851
SFS	Scf_2L	5720094	5723926
SFS	Scf_2L	6275183	6278257
SFS	Scf_2L	6405504	6414200
SFS	Scf_2L	7471873	7477916
SFS	Scf_2L	7841365	7853102
SFS	Scf_2L	7985462	7990300
SFS	Scf_2L	12447342	12453128
SFS	Scf_2L	12954618	12961122
SFS	Scf_2L	13146195	13149738
SFS	Scf_2L	14290375	14302075
SFS	Scf_2L	14436417	14450740
SFS	Scf_2L	16115525	16119094
SFS	Scf_2L	17192086	17196675
SFS	Scf_2L	18996291	19002683
SFS	Scf_2L	19661925	19665996
SFS	Scf_2L	19671935	19675299
SFS	Scf_2L	20527524	20540585
SFS	Scf_2L	20553626	20562308
SFS	Scf_2L	20564050	20573114
SFS	Scf_2L	20575003	20584644
SFS	Scf_2L	20599381	20609947
SFS	Scf_2L	20623883	20627635
SFS	Scf_2L	20652500	20663600
SFS	Scf_2L	20675665	20792935
SFS	Scf_2L	20819875	20834547
SFS	Scf_2L	20854486	20887212
SFS	Scf_2L	20893079	20924816
SFS	Scf_2L	20925035	20935729
SFS	Scf_2L	20938549	20959905
SFS	Scf_2L	20976749	21010539
SFS	Scf_2L	21018009	21028697
SFS	Scf_2L	21041145	21061582
SFS	Scf_2L	21072115	21079602
SFS	Scf_2L	21157835	21171478
SFS	Scf_2L	21197190	21205627
SFS	Scf_2L	21208226	21228423
SFS	Scf_2L	21239043	21259977
SFS	Scf_2L	21298178	21303999
SFS	Scf_2L	21332610	21346083
SFS	Scf_2L	21351011	21368630

SFS	Scf_2L	21380966	21392495
SFS	Scf_2L	21478312	21486319
SFS	Scf_2L	21495071	21515623
SFS	Scf_2L	21543448	21567529
SFS	Scf_2L	21571432	21581576
SFS	Scf_2L	21597588	21601989
SFS	Scf_2L	21609660	21618326
SFS	Scf_2L	21638943	21649282
SFS	Scf_2L	21685881	21700938
SFS	Scf_2L	21707642	21728920
SFS	Scf_2L	21752226	21765345
SFS	Scf_2L	21785989	21791528
SFS	Scf_2L	21811511	21824517
SFS	Scf_2L	21827911	21832044
SFS	Scf_2L	21960069	21973655
SFS	Scf_2L	21991064	21998382
SFS	Scf_2L	22028922	22032929
SFS	Scf_2L	22049909	22055794
SFS	Scf_2L	22058589	22066025
SFS	Scf_2L	22086710	22092706
SFS	Scf_2L	22099418	22105347
SFS	Scf_2L	22169057	22179292
SFS	Scf_2L	22190480	22201613
SFS	Scf_2L	22218878	22235817
SFS	Scf_2L	22251989	22264062
SFS	Scf_2L	22265684	22267450
SFS	Scf_2L	22314401	22325523
SFS	Scf_2L	22358741	22363533
SFS	Scf_2L	22385034	22393581
SFS	Scf_2L	22444228	22449815
SFS	Scf_2L	22470929	22477659
SFS	Scf_2L	22628510	22635999
SFS	Scf_2L	22988064	23009416
SFS	Scf_2L	23013322	23020776
SFS	Scf_2L	23114476	23127727
SFS	Scf_2L	23162330	23179729
SFS	Scf_2L	23265717	23276547
SFS	Scf_2L	23308755	23336334
SFS	Scf_2L	23361820	23365715
SFS	Scf_2L	23373153	23381184
SFS	Scf_2L	23406915	23417552
SFS	Scf_2L	23419290	23436757
SFS	Scf_2L	23455322	23459867
SFS	Scf_2L	23491548	23494369
SFS	Scf_2L	23523769	23533287
SFS	Scf_2R	10778	15648
SFS	Scf_2R	59116	69408
SFS	Scf_2R	181555	188104
SFS	Scf_2R	285827	296603
SFS	Scf_2R	372423	378474
SFS	Scf_2R	508807	514718
SFS	Scf_2R	567777	595354

SFS	Scf_2R	636724	647547
SFS	Scf_2R	655799	661407
SFS	Scf_2R	677286	687870
SFS	Scf_2R	725458	729654
SFS	Scf_2R	851752	858962
SFS	Scf_2R	949613	959408
SFS	Scf_2R	1041603	1051649
SFS	Scf_2R	1064967	1076035
SFS	Scf_2R	1108116	1142208
SFS	Scf_2R	1176978	1183390
SFS	Scf_2R	1187885	1198887
SFS	Scf_2R	1211907	1226898
SFS	Scf_2R	1269044	1285920
SFS	Scf_2R	1366798	1377332
SFS	Scf_2R	1402274	1408185
SFS	Scf_2R	1410363	1435988
SFS	Scf_2R	1452930	1464978
SFS	Scf_2R	1561748	1590709
SFS	Scf_2R	1615451	1639860
SFS	Scf_2R	1647369	1682047
SFS	Scf_2R	1684960	1693767
SFS	Scf_2R	1742527	1753891
SFS	Scf_2R	1773921	1778542
SFS	Scf_2R	1799056	1820836
SFS	Scf_2R	1824752	1833403
SFS	Scf_2R	2886690	2892761
SFS	Scf_2R	3151262	3161927
SFS	Scf_2R	3177407	3204652
SFS	Scf_2R	3218994	3225206
SFS	Scf_2R	3232140	3241795
SFS	Scf_2R	4513258	4519777
SFS	Scf_2R	4827811	4834127
SFS	Scf_2R	5639123	5659294
SFS	Scf_2R	6275233	6295054
SFS	Scf_2R	6536359	6563420
SFS	Scf_2R	6578111	6603807
SFS	Scf_2R	6609004	6612707
SFS	Scf_2R	6696547	6704536
SFS	Scf_2R	7347260	7351544
SFS	Scf_2R	8707934	8813749
SFS	Scf_2R	8865696	8876576
SFS	Scf_2R	8891554	8897074
SFS	Scf_2R	8911408	8924952
SFS	Scf_2R	9044713	9061145
SFS	Scf_2R	9276416	9281511
SFS	Scf_2R	9824174	9858825
SFS	Scf_2R	10395127	10405040
SFS	Scf_2R	10842699	10848087
SFS	Scf_2R	10889246	10896524
SFS	Scf_2R	11229464	11237779
SFS	Scf_2R	11241100	11249031
SFS	Scf_2R	11277844	11291040

SFS	Scf_2R	11400086	11406155
SFS	Scf_2R	11578393	11585352
SFS	Scf_2R	11794190	11801480
SFS	Scf_2R	11804111	11819599
SFS	Scf_2R	11865254	11880190
SFS	Scf_2R	13620871	13627911
SFS	Scf_2R	13895554	13924316
SFS	Scf_2R	14803625	14807955
SFS	Scf_2R	15365359	15370202
SFS	Scf_2R	16548556	16553073
SFS	Scf_2R	18653364	18659162
SFS	Scf_2R	18886919	18896940
SFS	Scf_2R	18898262	18907957
SFS	Scf_2R	19041046	19045855
SFS	Scf_2R	19589780	19593652
SFS	Scf_2R	19669875	19678222
SFS	Scf_2R	21221699	21227554
SFS	Scf_2R	21298624	21304863
SFS	Scf_2R	21392381	21420994
SFS	Scf_2R	21468645	21475710
SFS	Scf_2R	21533071	21544591
SFS	Scf_3L	39891	56354
SFS	Scf_3L	96078	133359
SFS	Scf_3L	153603	160527
SFS	Scf_3L	316810	320844
SFS	Scf_3L	533295	541562
SFS	Scf_3L	1970187	1980549
SFS	Scf_3L	2381835	2389719
SFS	Scf_3L	2410574	2416611
SFS	Scf_3L	2682820	2700220
SFS	Scf_3L	3024758	3046649
SFS	Scf_3L	3083571	3118322
SFS	Scf_3L	4522102	4529512
SFS	Scf_3L	4676258	4688590
SFS	Scf_3L	5323055	5333003
SFS	Scf_3L	5650063	5655038
SFS	Scf_3L	6029094	6032933
SFS	Scf_3L	6048573	6063467
SFS	Scf_3L	6124676	6129583
SFS	Scf_3L	6434510	6442120
SFS	Scf_3L	8075477	8112572
SFS	Scf_3L	9756128	9760059
SFS	Scf_3L	9901034	9905425
SFS	Scf_3L	11212422	11248302
SFS	Scf_3L	11252374	11261136
SFS	Scf_3L	12033790	12049346
SFS	Scf_3L	12074601	12079464
SFS	Scf_3L	12301117	12304382
SFS	Scf_3L	14959449	14973543
SFS	Scf_3L	16196637	16212516
SFS	Scf_3L	16259763	16266705
SFS	Scf_3L	16994149	17002236

SFS	Scf_3L	17155082	17161266
SFS	Scf_3L	17377583	17392470
SFS	Scf_3L	17411327	17421596
SFS	Scf_3L	17436862	17442038
SFS	Scf_3L	18362429	18370589
SFS	Scf_3L	18908419	18913942
SFS	Scf_3L	19386321	19400316
SFS	Scf_3L	19765908	19783486
SFS	Scf_3L	20901597	20912368
SFS	Scf_3L	20917860	20943217
SFS	Scf_3L	20959114	20967021
SFS	Scf_3L	21468316	21487335
SFS	Scf_3L	21735366	21746231
SFS	Scf_3L	22596625	22605313
SFS	Scf_3L	22672755	22676948
SFS	Scf_3L	22681418	22693251
SFS	Scf_3L	22715127	22750117
SFS	Scf_3L	22751833	22753599
SFS	Scf_3L	22857485	22864845
SFS	Scf_3L	23084675	23090421
SFS	Scf_3L	23157461	23163671
SFS	Scf_3L	23327175	23330162
SFS	Scf_3L	23373014	23381811
SFS	Scf_3L	23489958	23493123
SFS	Scf_3L	23521734	23528933
SFS	Scf_3L	23731180	23736891
SFS	Scf_3L	23753216	23768613
SFS	Scf_3L	23983884	23991203
SFS	Scf_3L	24115154	24121817
SFS	Scf_3R	109084	115127
SFS	Scf_3R	1971331	1995262
SFS	Scf_3R	3479478	3509815
SFS	Scf_3R	3783005	3787712
SFS	Scf_3R	4498558	4505776
SFS	Scf_3R	4851306	4859380
SFS	Scf_3R	9120106	9157107
SFS	Scf_3R	11758417	11770969
SFS	Scf_3R	11774180	11781481
SFS	Scf_3R	11807153	11834934
SFS	Scf_3R	11854263	11859675
SFS	Scf_3R	11867627	11874556
SFS	Scf_3R	11900702	11904845
SFS	Scf_3R	11978473	11988951
SFS	Scf_3R	11999386	12003456
SFS	Scf_3R	12048762	12085649
SFS	Scf_3R	12270654	12275546
SFS	Scf_3R	13183627	13191076
SFS	Scf_3R	13412471	13416657
SFS	Scf_3R	14738991	14747744
SFS	Scf_3R	17069068	17075728
SFS	Scf_3R	17426951	17432830
SFS	Scf_3R	19216382	19220692

SFS	Scf_3R	19517707	19527350
SFS	Scf_3R	19531697	19538083
SFS	Scf_3R	19553509	19559854
SFS	Scf_3R	20101220	20105309
SFS	Scf_3R	20793218	20809366
SFS	Scf_3R	23075089	23085146
SFS	Scf_3R	23091976	23099121
SFS	Scf_3R	24158627	24163371
SFS	Scf_3R	24299715	24304693
SFS	Scf_3R	25283856	25305570
SFS	Scf_3R	25589826	25600813
SFS	Scf_3R	25724968	25731347
SFS	Scf_3R	26185952	26191821
SFS	Scf_3R	26965697	26974453
SFS	Scf_3R	27021553	27063066
SFS	Scf_3R	27120278	27151399
SFS	Scf_X	10247	97984
SFS	Scf_X	98453	347357
SFS	Scf_X	444194	470408
SFS	Scf_X	592927	624798
SFS	Scf_X	967616	977141
SFS	Scf_X	1104776	1117106
SFS	Scf_X	1380304	1386855
SFS	Scf_X	1419470	1449531
SFS	Scf_X	1484437	1521455
SFS	Scf_X	1527596	1552390
SFS	Scf_X	1621667	1696799
SFS	Scf_X	1755223	1786354
SFS	Scf_X	2022188	2028120
SFS	Scf_X	2030721	2039212
SFS	Scf_X	2665116	2672254
SFS	Scf_X	3155778	3162920
SFS	Scf_X	3375847	3397473
SFS	Scf_X	3513622	3531244
SFS	Scf_X	3537180	3551654
SFS	Scf_X	3653699	3674969
SFS	Scf_X	3861353	3878074
SFS	Scf_X	5502299	5523626
SFS	Scf_X	5741837	5756596
SFS	Scf_X	6016101	6039735
SFS	Scf_X	6116022	6123419
SFS	Scf_X	6140439	6148690
SFS	Scf_X	6175603	6185079
SFS	Scf_X	6345828	6356276
SFS	Scf_X	6526082	6542348
SFS	Scf_X	6641530	6649467
SFS	Scf_X	6988890	7008992
SFS	Scf_X	7069740	7077178
SFS	Scf_X	7111774	7121791
SFS	Scf_X	8441590	8450136
SFS	Scf_X	8455695	8462252
SFS	Scf_X	8462773	8468079

SFS	Scf_X	8648817	8689888
SFS	Scf_X	8692564	8818171
SFS	Scf_X	8837148	8872630
SFS	Scf_X	8950968	8959761
SFS	Scf_X	9033793	9064628
SFS	Scf_X	9110701	9163940
SFS	Scf_X	10128273	10139307
SFS	Scf_X	11493338	11498210
SFS	Scf_X	12374351	12390674
SFS	Scf_X	12690127	12710410
SFS	Scf_X	13166841	13178304
SFS	Scf_X	13437311	13459675
SFS	Scf_X	13466314	13472792
SFS	Scf_X	13519252	13530186
SFS	Scf_X	13907245	13914897
SFS	Scf_X	14053883	14066438
SFS	Scf_X	14301731	14373605
SFS	Scf_X	14696045	14703462
SFS	Scf_X	14911199	14940286
SFS	Scf_X	15462896	15469239
SFS	Scf_X	15914219	15945599
SFS	Scf_X	15954784	15982351
SFS	Scf_X	16065270	16073828
SFS	Scf_X	16221948	16237871
SFS	Scf_X	16242545	16265511
SFS	Scf_X	16366866	16405124
SFS	Scf_X	16555600	16576666
SFS	Scf_X	17300088	17320648
SFS	Scf_X	17323157	17333784
SFS	Scf_X	17600365	17612244
SFS	Scf_X	17612574	17623744
SFS	Scf_X	18627065	18632443
SFS	Scf_X	18658568	18666583
SFS	Scf_X	19366417	19379019
SFS	Scf_X	19523303	19530790
SFS	Scf_X	19727689	19728431
SFS	Scf_X	19760203	19768092
SFS	Scf_X	19812521	19818410
SFS	Scf_X	20309973	20316559
SFS	Scf_X	20742283	20753712
SFS	Scf_X	20827336	20829645

Table S6. GO enrichment analysis of genes within selective sweep regions in the 2018 data.

Slope	Category	Term	Count	PValue	Genes	FDR (%)
NFS	GOTERM_CC_	GO:0005737~cytoplasm	59	9.53E-04	RL, OCRL, LST8, (	1.206617086
NFS	KEGG_PATHW	dme04141:Protein processing in endoplasmic reticu	9	0.00478	4AB, L(1)G0320,	4.511750621
NFS	GOTERM_BP_	GO:0001555~oocyte growth	3	0.00496	CB, ITGAPS4, JAG	7.316492885
NFS	GOTERM_CC_	GO:0090575~RNA polymerase II transcription facto	3	0.01216	AC, SC, ASE	14.41829192
NFS	GOTERM_BP_	GO:0046701~insecticide catabolic process	4	0.01485	CYP6G1, CYP6G2	20.44288944
NFS	GOTERM_CC_	GO:0008305~integrin complex	3	0.01594	, ITGAPS4, MSP3	18.50050491
NFS	GOTERM_BP_	GO:0061382~Malpighian tubule tip cell differentiati	3	0.01681	AC, SC, ASE	22.82181289
NFS	GOTERM_BP_	GO:0045886~negative regulation of synaptic growt	5	0.01899	EWG, STJ, SKPA,	25.39773451
NFS	GOTERM_BP_	GO:0008407~chaeta morphogenesis	5	0.02043	C, SC, F, JAGN, AS	27.0609178
NFS	GOTERM_BP_	GO:0007614~short-term memory	4	0.02074	BO, SCB, G9A, KI	27.40945563
NFS	GOTERM_BP_	GO:0007362~terminal region determination	4	0.02074	L, FS(1)M3, PEB,	27.40945563
NFS	GOTERM_BP_	GO:0045746~negative regulation of Notch signaling	5	0.02353	AP160, KRZ, PIGS	30.50025559
NFS	GOTERM_CC_	GO:0030529~intracellular ribonucleoprotein comple	4	0.024	2, NBR, LARP7, H	26.59418473
NFS	GOTERM_BP_	GO:0007616~long-term memory	6	0.02413	E, SVR, GRY, ARM	31.15981341
NFS	GOTERM_BP_	GO:0046680~response to DDT	4	0.02528	CYP6G1, CYP6G2	32.38501519
NFS	GOTERM_MF_	GO:0003676~nucleic acid binding	18	0.02747	CG10979, CG188	31.0051876
NFS	GOTERM_MF_	GO:0000166~nucleotide binding	11	0.02933	RP7, DAP160, CC	32.73991814
NFS	GOTERM_CC_	GO:0048471~perinuclear region of cytoplasm	6	0.03277	, MBL, WASH, M	34.56378424
NFS	GOTERM_MF_	GO:0005515~protein binding	24	0.03279	M5, RAB19, DOR,	35.86131494
NFS	GOTERM_BP_	GO:0040011~locomotion	4	0.03582	, MSP300, KIS, H	42.73999771
NFS	GOTERM_BP_	GO:0007417~central nervous system development	6	0.03928	, EWG, ASE, SMI	45.79884265
NFS	GOTERM_BP_	GO:0017085~response to insecticide	3	0.03942	6G1, CYP6G2, GR	45.92108968
NFS	GOTERM_BP_	GO:0035075~response to ecdysone	3	0.03942	USP, TRR, HFW	45.92108968
NFS	GOTERM_BP_	GO:0050767~regulation of neurogenesis	3	0.03942	AC, SC, ASE	45.92108968
NFS	KEGG_PATHW	dme04350:TGF-beta signaling pathway	4	0.04051	LA, BABO, SKPA,	32.85316233
NFS	GOTERM_BP_	GO:0007517~muscle organ development	6	0.04105	P, MBL, FRG1, SI	47.30675989
NFS	GOTERM_BP_	GO:0007419~ventral cord development	6	0.04287	ODY, AC, SC, NOI	48.81444366
SFS	GOTERM_CC_	GO:0090575~RNA polymerase II transcription facto	5	7.05E-05	SC, L(1)SC, ASE, H	0.092323943
SFS	GOTERM_BP_	GO:0007517~muscle organ development	12	1.25E-04	LTA, FRG1, SU(Z)	0.198967546
SFS	GOTERM_BP_	GO:0045214~sarcomere organization	7	4.81E-04	NB2, SCGDELTA, S	0.76014791
SFS	GOTERM_BP_	GO:0007417~central nervous system development	11	5.02E-04	ASE, ELAV, CT, KU	0.793969376
SFS	GOTERM_BP_	GO:0045944~positive regulation of transcription fro	19	9.39E-04	EF, ARM, HR39, I	1.479939809
SFS	GOTERM_BP_	GO:0045746~negative regulation of Notch signaling	8	0.00121	P160, KRZ, PIGS,	1.898092171
SFS	GOTERM_MF_	GO:0005096~GTPase activator activity	11	0.00145	182, CONU, RHO(	1.98577343
SFS	GOTERM_BP_	GO:0007165~signal transduction	15	0.00229	13F, CONU, BAB	3.573753645
SFS	GOTERM_BP_	GO:0061382~Malpighian tubule tip cell differentiati	4	0.00294	C, SC, L(1)SC, AS	4.572700221
SFS	GOTERM_BP_	GO:0010906~regulation of glucose metabolic proce	15	0.00332	MD3, HR39, MON	5.135814492
SFS	GOTERM_BP_	GO:0050907~detection of chemical stimulus involv	4	0.00714	A, IR75A, IR75B,	10.74880392
SFS	GOTERM_MF_	GO:0003700~transcription factor activity, sequence	20	0.0074	74EF, DSF, SR, M	9.784791698
SFS	GOTERM_CC_	GO:0005737~cytoplasm	79	0.00762	PC, CG32165, CG	9.540381596
SFS	GOTERM_CC_	GO:0005886~plasma membrane	45	0.00801	PARA, L(2)GL, S(	10.00818489
SFS	GOTERM_BP_	GO:0007419~ventral cord development	9	0.00836	C, NOLO, DIMM	12.47381765
SFS	GOTERM_BP_	GO:0007525~somatic muscle development	6	0.00842	HOW, TPI, SLS, S	12.55967537
SFS	GOTERM_BP_	GO:0007016~cytoskeletal anchoring at plasma mer	4	0.00905	A, P130CAS, ARI	13.43072696
SFS	GOTERM_BP_	GO:0008586~imaginal disc-derived wing vein morp	7	0.00955	EP, NET, TAY, HE	14.12420381
SFS	GOTERM_CC_	GO:0030018~Z disc	6	0.01055	IP, FRG1, TPI, SLS	12.97675039
SFS	KEGG_PATHW	dme00630:Glyoxylate and dicarboxylate metabolis	5	0.01055	T, CG7430, CG55	10.42535952
SFS	GOTERM_BP_	GO:0017085~response to insecticide	4	0.01122	L, CYP6G2, GR66	16.39915017

SFS	GOTERM_BP_	GO:0050767~regulation of neurogenesis	4	0.01122	.C, SC, L(1)SC, AS	16.39915017
SFS	GOTERM_BP_	GO:0040018~positive regulation of multicellular org	7	0.01138	'EP, DSOR1, MI, (	16.61682222
SFS	GOTERM_BP_	GO:0007400~neuroblast fate determination	5	0.01149	SC, L(1)SC, ASE, I	16.75967942
SFS	GOTERM_CC_	GO:0005667~transcription factor complex	7	0.01218	EWG, WASH, L(1	14.84714752
SFS	GOTERM_CC_	GO:0030425~dendrite	11	0.01353	2A, OR22B, GR8A	16.35458932
SFS	GOTERM_BP_	GO:0007220~Notch receptor processing	4	0.01367	MX, DOR, KUZ, D	19.62907073
SFS	GOTERM_BP_	GO:0000122~negative regulation of transcription fr	12	0.01423	TD, LZ, CT, KIS, C	20.34855802
SFS	GOTERM_CC_	GO:0071683~sensory dendrite	4	0.01523	N, IR75A, OR22A	18.22538533
SFS	GOTERM_MF_	GO:0008134~transcription factor binding	9	0.01578	, GCE, BTD, H, AR	19.79606246
SFS	GOTERM_MF_	GO:0003707~steroid hormone receptor activity	4	0.01686	P, DSF, HR39, HR	21.00637081
SFS	GOTERM_MF_	GO:0004879~RNA polymerase II transcription facto	4	0.02018	P, DSF, HR39, HR	24.63383462
SFS	GOTERM_BP_	GO:0008407~chaeta morphogenesis	6	0.02059	C, SC, F, ASE, B, J	28.12460305
SFS	GOTERM_BP_	GO:0006351~transcription, DNA-templated	22	0.0207	SF, MAF1, H, HR3	28.25160794
SFS	GOTERM_BP_	GO:0043401~steroid hormone mediated signaling p	4	0.02272	P, DSF, HR39, HR	30.56286906
SFS	KEGG_PATHW	dme04350:TGF-beta signaling pathway	5	0.02834	, BABO, SKPA, RB	25.80116861
SFS	GOTERM_BP_	GO:0043087~regulation of GTPase activity	6	0.02848	G8155, CG6182, (	36.78592793
SFS	GOTERM_BP_	GO:0045931~positive regulation of mitotic cell cycle	3	0.03651	MI, SKPA, MCM1	44.58791448
SFS	GOTERM_BP_	GO:0043277~apoptotic cell clearance	3	0.03651	3, CG18132, SRC4	44.58791448
SFS	GOTERM_CC_	GO:0005794~Golgi apparatus	12	0.03661	9, CERT, INTS3, C	38.67667082
SFS	GOTERM_BP_	GO:0007399~nervous system development	9	0.04184	V, ALP4, H, ARM,	49.25393635
SFS	GOTERM_MF_	GO:0045296~cadherin binding	3	0.04214	DN, P120CTN, AF	44.96620607
SFS	GOTERM_BP_	GO:0006468~protein phosphorylation	16	0.04281	, STLK, BABO, SA	50.06738283
SFS	GOTERM_BP_	GO:0016337~single organismal cell-cell adhesion	4	0.04345	20CTN, P130CAS	50.59795526
SFS	GOTERM_BP_	GO:0045995~regulation of embryonic developmen	3	0.04461	OP1, HOW, WNT	51.53973765
SFS	GOTERM_BP_	GO:0007519~skeletal muscle tissue development	3	0.04461	KUZ, MEF2, SLS	51.53973765
SFS	GOTERM_CC_	GO:0000137~Golgi cis cisterna	3	0.04491	GLG1, GARZ, LVA	45.25100736
SFS	GOTERM_MF_	GO:0000978~RNA polymerase II core promoter pro	4	0.0471	EWG, BI, H, HR39	48.79108289
SFS	GOTERM_MF_	GO:0005328~neurotransmitter:sodium symporter a	4	0.0471	, CG13795, BLOT	48.79108289
SFS	GOTERM_BP_	GO:0006836~neurotransmitter transport	4	0.04843	, CG13795, BLOT	54.52319304

Table S7. Counts of co-occurring major alleles between D. simulans and D. melanogaster across all trans-species polymorphic sites.

Category	*	# of sites (compare between D. sim and D. mel in same slope) in	
		NFS	SFS
ALL sites	S.A	31613	32318
	D.A	11820	11115
Sites in CDS	S.A	4058	4126
	D.A	1643	1575
Non-synonymous sites	S.A	551	567
	D.A	186	170

*: S.A (Same allele); D.A (Different allele)

Table S8. Correlation of changes of frequency of two slopes for trans-species polymorphic sites between D. simulans and D. melanogaster.

Regions	# of site	r (spearman)	p-value
**3-UTR**	1,394	0.01478	0.5813
**5-UTR**	967	-0.00384	0.9051
**CDS**	5,701	0.01647	0.2137
**non-synonymous**	737	0.06570	0.07466
**synonymous**	4,974	0.00987	0.4861
**intergenic**	13,671	0.00853	0.3188
**intron**	20,856	-0.00563	0.4161
**promoter**	441	0.04818	0.3128
**ncRNA**	403	0.10541	0.03443
**Total**	43,433	0.00400	0.4041

Table S9. Distribution of TEs across chromosomal arms.

Slope	Chr/Arm	Chr_size	3-UTR	5-UTR	CDS	Intergenic	Intron	Promoter	Total	TE density (per 10 kb)
**NFS**	Scf_2L	23,539,531	50	51	103	927	820	24	**1,975**	0.84
	Scf_2R	21,544,594	58	51	96	863	886	19	**1,973**	0.92
	Scf_3L	24,153,973	58	45	112	809	748	27	**1,799**	0.74
	Scf_3R	27,160,941	66	58	109	596	795	24	**1,648**	0.61
	Scf_4	1,026,345	1	1	5	75	158	1	**241**	2.35
	Scf_X	20,829,647	49	28	94	598	614	17	**1,400**	0.67
	**Total**	**118,255,031**	**282**	**234**	**519**	**3,868**	**4,021**	**112**	**9,036**	0.76
**SFS**	Scf_2L	23,539,531	29	56	93	961	802	28	**1,969**	0.84
	Scf_2R	21,544,594	66	53	105	859	894	16	**1,993**	0.93
	Scf_3L	24,153,973	62	62	117	810	736	26	**1,813**	0.75
	Scf_3R	27,160,941	64	68	113	604	783	30	**1,662**	0.61
	Scf_4	1,026,345	2	0	5	71	163	4	**245**	2.39
	Scf_X	20,829,647	43	40	99	636	657	25	**1,500**	0.72
	**Total**	**118,255,031**	**266**	**279**	**532**	**3,941**	**4,035**	**129**	**9,182**	0.78

Table S10. Number of TEs in each family for NFS and SFS.

TEFamily	NumNFS	NumSFS	P-value (Fisher Exact Test)
roo	610	554	0.048965653
hobo	1370	1298	0.053842219
P-element	285	336	0.06042645
ninja	18	32	0.064953513
Tabor	49	70	0.066399477
mariner	69	93	0.082272996
opus	33	23	0.181477385
baggins	77	95	0.22032439
G-element	63	53	0.35167097
transib	542	580	0.371606128
1731	21	16	0.414066333
Quasimodo	8	5	0.420266985
Max-element	270	256	0.426135988
Cr1a	283	307	0.426668241
1360	452	483	0.439989581
micropia	22	18	0.529519718
copia	4	7	0.548843366
BS	32	38	0.550508514
GATE	76	70	0.561896936
Doc	314	304	0.566694195
Isfun-1	23	28	0.575916137
Rt	171	184	0.59234595
HMS-Beagle	27	32	0.602996698
flea	49	44	0.603562364
I-element	250	242	0.615174117
G	179	191	0.636849987
Circe	24	21	0.656254609
Kepler	21	25	0.658788537
hopper	66	73	0.670463554
3S18	24	28	0.677862955
HeT-A	2	4	0.687506327
springer	11	14	0.690063193
jockey	83	79	0.693726655
X-element	90	97	0.71348815
FB	147	143	0.722756463
Minos	4	3	0.724420468
TART	218	214	0.733356875
Idefix	17	20	0.742860858
Tom	20	18	0.747367592
aurora-element	5	4	0.751979886
Stalker	22	25	0.770902955
R1-2	6	5	0.772649574
412	160	168	0.780707083
Bari1	26	29	0.787745388
Helena	133	131	0.804505068
mdg	50	48	0.839631078
diver	205	213	0.843138619
mini-me	12	14	0.845032655

INE-1	957	981	0.847556857
F-element	14	13	0.849392249
gtwin	13	15	0.850568225
Porto1	15	14	0.854413378
S-element	15	17	0.860065286
R1A1-element	19	21	0.874646393
blood	33	35	0.903619847
Ivk	35	34	0.904247989
McClintock	37	36	0.906854996
HB	165	170	0.912250912
Tc	254	256	0.928502781
17.6	4	4	1
297	61	61	1
Burdock	51	52	1
Dm88	6	6	1
Fw	10	10	1
GEM	1	1	1
Juan	324	330	1
NOF	1	1	1
Osvaldo	6	6	1
Q-element	1	1	1
R2-element	2	3	1
S2	4	4	1
SGM	3	3	1
TART-C	3	3	1
Transpac	15	16	1
ZAM	1	1	1
accord	86	87	1
frogger	1	2	1
gypsy	159	162	1
invader	63	64	1
looper	3	3	1
rover	30	31	1

Table S11. List of TE insertions with extreme TE scores (|score|>=6).

#CHR	POS	TE_FAMILY	TE_ORDER	TE_SCORE	GENEID	GENESYMBOL	REGION
Scf_3L	7298365	INE-1	TIR	-8	FBgn0185712;FBgn0184776	CG42458;sphinx2	3-UTR
Scf_3L	8164590	HB	TIR	7	-		intergenic
Scf_3R	295769	INE-1	TIR	6	FBgn0191164	CG34357	intron
Scf_3L	24086149	3S18	LTR	6	-		intergenic
Scf_X	20765441	1360	TIR	6	-		intergenic
Scf_X	19380457	INE-1	TIR	6	FBgn0195985	shakB	intron
Scf_2L	17336387	hobo	TIR	6	FBgn0193270	CadN2	intron
Scf_2L	5440005	hobo	TIR	6	FBgn0194037	DIP	intron
Scf_X	20792565	Tc1	TIR	-6	-		intergenic
Scf_3L	18161628	FB	foldback	-6	FBgn0186435	MYPT	intron
Scf_3R	2871334	HB	TIR	-6	FBgn0190999	Alh	intron
Scf_3L	18011155	1360	TIR	-6	-		intergenic
Scf_3L	18491043	1360	TIR	-6	-		intergenic
Scf_3L	799	INE-1	TIR	-6	-		intergenic
Scf_3L	23072087	hopper	TIR	-6	FBgn0269968	CG40470	intron
Scf_3L	11450988	hobo	TIR	-6	FBgn0184506	CG6024	intron
Scf_2L	21648509	transib4	TIR	5	-		intergenic

Table S12. List of insertions within the CDS regions per TE families 412, P-element, mariner and G-element.

#CHR	POS	TE_FAMILY	TE_ORDER	TE_SCORE	GENE_ID	GENE_SYMBOL	REGION
Scf_2R	9682695	412	LTR	3	FBgn0182702	CG13325	CDS
Scf_X	8479174	P-element	TIR	2	FBgn0270774;FBgn0188502	FBgn0270774;Nost	CDS
Scf_2L	19933051	412	LTR	2	FBgn0268401	FBgn0268401	CDS
Scf_3L	14791649	mariner	TIR	2	FBgn0184296	CG7011	CDS
Scf_3L	6174742	mariner	TIR	-2	FBgn0268606	FBgn0268606	CDS
Scf_X	18648566	P-element	TIR	-1	FBgn0270786;FBgn0268400	Hers;amn	CDS
Scf_2R	15250355	412	LTR	-1	FBgn0196658	Jabba	CDS
Scf_3R	18730785	P-element	TIR	1	FBgn0269882;FBgn0189308	FBgn0269882;CG44(	CDS
Scf_2L	21974482	mariner	TIR	1	FBgn0269536	His2A:CG33853	CDS
Scf_2R	12054104	412	LTR	1	FBgn0268495	Ir52b	CDS
Scf_2R	5792543	P-element	TIR	1	FBgn0268246	FBgn0268246	CDS
Scf_2R	16749232	P-element	TIR	1	FBgn0196592	MED8	CDS
Scf_2L	19697662	412	LTR	1	FBgn0195597	FBgn0195597	CDS
Scf_2L	16683993	G-element	non-LTR	1	FBgn0195455;FBgn0195456	beat;CG34106	CDS
Scf_2L	8104251	P-element	TIR	1	FBgn0194911	CG17294	CDS
Scf_2L	4650349	G-element	non-LTR	1	FBgn0194086	Fnta	CDS
Scf_3R	8315298	P-element	TIR	1	FBgn0191728	cher	CDS
Scf_3R	7826707	G-element	non-LTR	1	FBgn0191699	CG14323	CDS
Scf_3R	17036084	P-element	TIR	1	FBgn0190036	puc	CDS
Scf_3R	18908004	mariner	TIR	1	FBgn0189903	CG16732	CDS
Scf_3R	20441653	mariner	TIR	1	FBgn0189770	CG34027	CDS
Scf_X	19960272	P-element	TIR	1	FBgn0189083;FBgn0270445	bbx;FBgn0270445	CDS
Scf_3L	21632214	412	LTR	1	FBgn0186694;FBgn0270422	olf413;FBgn0270422	CDS
Scf_3L	7888295	mariner	TIR	1	FBgn0185744;FBgn0268446	Ect4;FBgn0268446	CDS
Scf_3L	1239986	G-element	non-LTR	1	FBgn0185318	Sherpa	CDS
Scf_3L	9146265	P-element	TIR	1	FBgn0184657	Hsp67Ba	CDS
Scf_3L	16294161	P-element	TIR	1	FBgn0184179;FBgn0268771	Lasp;FBgn0268771	CDS
Scf_2R	14664011	P-element	TIR	1	FBgn0183090	pAbp	CDS
Scf_2R	8958113	mariner	TIR	1	FBgn0182628	CG30043	CDS
Scf_3R	20108724	P-element	TIR	-1	FBgn0270540	FBgn0270540	CDS
Scf_3L	605265	P-element	TIR	-1	FBgn0270043	FBgn0270043	CDS
Scf_3L	2215978	mariner	TIR	-1	FBgn0269913	FBgn0269913	CDS
Scf_X	4423104	P-element	TIR	-1	FBgn0269796;FBgn0188299	FBgn0269796;CG42!	CDS
Scf_3L	9149809	P-element	TIR	-1	FBgn0268830;FBgn0185863	FBgn0268830;Hsp23	CDS
Scf_3R	19727915	412	LTR	-1	FBgn0268360	CG13614	CDS
Scf_2R	12781894	412	LTR	-1	FBgn0196843	CG15706	CDS
Scf_2L	20238438	P-element	TIR	-1	FBgn0195631	CG9331	CDS
Scf_2L	9753771	mariner	TIR	-1	FBgn0195033	Spn31A	CDS
Scf_2L	58391	P-element	TIR	-1	FBgn0194390	galectin	CDS
Scf_2L	21031599	412	LTR	-1	FBgn0193054	CG31612	CDS
Scf_3R	6669752	mariner	TIR	-1	FBgn0191630	CG7720	CDS
Scf_3R	2853079	P-element	TIR	-1	FBgn0191002;FBgn0269480	Alh;FBgn0269480	CDS
Scf_3R	10616041	mariner	TIR	-1	FBgn0190482;FBgn0270218	jvl;FBgn0270218	CDS
Scf_3R	15847264	P-element	TIR	-1	FBgn0190128;FBgn0269867	CG45050;FBgn0269{	CDS
Scf_3R	18906078	mariner	TIR	-1	FBgn0189904	CG10170	CDS
Scf_3R	22892750	G-element	non-LTR	-1	FBgn0189611	tau	CDS
Scf_X	4362430	412	LTR	-1	FBgn0188294	CG6978	CDS
Scf_3R	26847146	P-element	TIR	-1	FBgn0187960	awd	CDS

Scf_X	17984220	P-element	TIR	-1	FBgn0187262	CG7990	CDS
Scf_2R	4879142	P-element	TIR	-1	FBgn0187036	LRP1	CDS
Scf_3R	22507008	G-element	non-LTR	-1	FBgn0186858	DNApol	CDS
Scf_3R	736933	412	LTR	-1	FBgn0186793	CG14662	CDS
Scf_3L	13516583	412	LTR	-1	FBgn0186141	Hml	CDS
Scf_3L	6078267	412	LTR	-1	FBgn0185641	CG7376	CDS
Scf_3L	7746038	P-element	TIR	-1	FBgn0184751	syd	CDS
Scf_3L	11858383	412	LTR	-1	FBgn0184470	Rh7	CDS
Scf_3L	11860235	412	LTR	-1	FBgn0184470	Rh7	CDS
Scf_2R	21186665	P-element	TIR	-1	FBgn0183645;FBgn0183646	CG44247;NKAIN	CDS
Scf_2R	18043376	P-element	TIR	-1	FBgn0183350;FBgn0196493	CG10082;CG30284	CDS
Scf_2R	17476985	P-element	TIR	-1	FBgn0183301	Treh	CDS
Scf_2R	15493299	412	LTR	-1	FBgn0183175	tn	CDS
Scf_2R	13070741	G-element	non-LTR	-1	FBgn0182955;FBgn0269463	Sema2a;FBgn02694	CDS
Scf_2R	12830416	412	LTR	-1	FBgn0182932	CG33017	CDS
Scf_2R	5841985	P-element	TIR	-1	FBgn0182404	Prp38	CDS
Scf_2R	3414928	P-element	TIR	-1	FBgn0182186;FBgn0269285	Adf1;FBgn0269285	CDS
Scf_X	15322283	P-element	TIR	0	FBgn0191705	Cyp1	CDS

Table S13. List of TEs found in 2018 data.

#TEFamily	NumNFS	NumSFS	Shared (#)	Shared (%)
INE-1	966	959	833	76.28
TART	265	273	219	68.65
HB	192	196	153	65.11
FB	190	190	127	50.2
hopper	77	73	50	50
Tc	331	321	206	46.19
BS	48	65	34	43.04
jockey	110	111	63	39.87
1360	678	733	377	36.46
Helena	158	169	79	31.85
invader	133	121	56	28.28
Ivk	50	63	24	26.97
Burdock	98	86	39	26.9
gypsy	274	273	111	25.46
transib	825	811	315	23.85
mdg	115	124	45	23.2
Circe	57	61	19	19.19
flea	81	99	27	17.65
Juan	502	471	140	16.81
3S18	64	72	19	16.24
blood	70	70	19	15.7
X-element	281	305	77	15.13
hobo	1735	1830	432	13.79
McClintock	128	153	34	13.77
Tabor	98	126	27	13.71
roo	1174	1294	272	12.39
I-element	568	783	140	11.56
micropia	75	62	14	11.38
HMS-Beagle	102	85	18	10.65
G	500	508	88	9.57
opus	81	80	13	8.78
accord	154	160	25	8.65
Idefix	101	86	14	8.09
Rt	680	760	102	7.62
rover	55	45	7	7.53
P-element	431	395	57	7.41
G-element	48	54	7	7.37
297	407	476	58	7.03
mariner	100	102	13	6.88
diver	1394	1632	178	6.25
R1A1-element	117	88	11	5.67
F-element	181	183	19	5.51
Doc	869	880	90	5.42
baggins	590	679	65	5.4
Cr1a	2238	2856	229	4.71
412	446	522	39	4.2
Max-element	1513	1630	102	3.35
GATE	1368	1663	36	1.2

Table S14. List of TE insertions in CDS regions of mating behavior-related genes from 2018 data.

Slope	Chromosome	InsertionPos	TEFamily	TEOrder	GeneID	GeneSymbol	Region
NFS	Scf_2L	11464203	Max-element	LTR	FBgn0193565;FBgn0193563	Pde1c;esc	CDS
NFS	Scf_2L	11484388	X-element	LINE	FBgn0193565;FBgn0195163	Pde1c;Mal	CDS
NFS	Scf_2L	16692658	roo	LTR	FBgn0195459	Gr36c	CDS
NFS	Scf_2L	17485391	GATE	LTR	FBgn0193269	btv	CDS
NFS	Scf_2L	20694343	opus	LTR	FBgn0193065;FBgn0195662	Mondo;Gr39a	CDS
NFS	Scf_2L	20697952	flea	LTR	FBgn0193065;FBgn0195662;	Mondo;Gr39a;	CDS
NFS	Scf_2R	3705649	Max-element	LTR	FBgn0182063;FBgn0182061;	mim;CheB42c;	CDS
NFS	Scf_2R	7959811	412	LTR	FBgn0187062	Gr47b	CDS
NFS	Scf_2R	12643581	TART	non-LTR	FBgn0196857	Dg	CDS
NFS	Scf_2R	12687273	Tc1	TIR	FBgn0182918;FBgn0270363	spin	CDS
NFS	Scf_2R	18015225	Cr1a	non-LTR	FBgn0196495	clt	CDS
NFS	Scf_2R	21400206	R1A1-element	non-LTR	FBgn0196212	lov	CDS
NFS	Scf_3L	3785596	Rt1c	non-LTR	FBgn0189152	Gr63a	CDS
NFS	Scf_3L	5132991	Doc	non-LTR	FBgn0184928;FBgn0270213	shep	CDS
NFS	Scf_3L	6584537	invader3	LTR	FBgn0184837	ple	CDS
NFS	Scf_3L	8103758	Rt1c	non-LTR	FBgn0184725	Gr66a	CDS
NFS	Scf_3L	8104301	412	LTR	FBgn0184725	Gr66a	CDS
NFS	Scf_3L	8104724	Max-element	LTR	FBgn0184725	Gr66a	CDS
NFS	Scf_3L	8877622	GATE	LTR	FBgn0269171	bol	CDS
NFS	Scf_3R	4201376	G2	non-LTR	FBgn0191473	SIFaR	CDS
NFS	Scf_3R	4201514	Cr1a	non-LTR	FBgn0191473	SIFaR	CDS
NFS	Scf_3R	4782096	GATE	LTR	FBgn0191523	Oamb	CDS
NFS	Scf_3R	13890061	TART	non-LTR	FBgn0025030	pros	CDS
NFS	Scf_3R	13890969	diver2	LTR	FBgn0025030	pros	CDS
NFS	Scf_3R	13891467	TART	non-LTR	FBgn0025030	pros	CDS
NFS	Scf_3R	15012370	roo	LTR	FBgn0190194	Syn	CDS
NFS	Scf_3R	20189753	diver2	LTR	FBgn0192588	Esp	CDS
NFS	Scf_3R	21212471	rooA	LTR	FBgn0192691	Lnk	CDS
NFS	Scf_3R	23983394	GATE	LTR	FBgn0192817;FBgn0192816	CheB98a;intr	CDS
NFS	Scf_X	2928910	Rt1c	non-LTR	FBgn0188231;FBgn0270899	dnc;dnc	CDS
NFS	Scf_X	5018708	GATE	LTR	FBgn0188321	SPR	CDS
NFS	Scf_X	11190287	Tc1	TIR	FBgn0187602	cac	CDS
NFS	Scf_X	12167788	Doc	non-LTR	FBgn0188661;FBgn0268256	fne	CDS
NFS	Scf_X	13866647	opus	LTR	FBgn0083415	rut	CDS
NFS	Scf_X	18638406	P-element	TIR	FBgn0270786;FBgn0268400	Hers;amn	CDS
NFS	Scf_X	18645589	transib2	TIR	FBgn0270786;FBgn0268400	Hers;amn	CDS
NFS	Scf_X	18646738	roo	LTR	FBgn0270786;FBgn0268400	Hers;amn	CDS
NFS	Scf_X	18647921	roo	LTR	FBgn0270786;FBgn0268400	Hers;amn	CDS
NFS	Scf_X	18648483	P-element	TIR	FBgn0270786;FBgn0268400	Hers;amn	CDS
NFS	Scf_X	18648695	P-element	TIR	FBgn0270786;FBgn0268400	Hers;amn	CDS
NFS	Scf_X	20361580	diver2	LTR	FBgn0182098	tilB	CDS
SFS	Scf_2L	1719514	I-element	non-LTR	FBgn0189784	Gr22a	CDS
SFS	Scf_2L	1719762	Cr1a	non-LTR	FBgn0189784	Gr22a	CDS
SFS	Scf_2L	4880510	transib2	TIR	FBgn0185902	qtc	CDS
SFS	Scf_2L	7207416	Cr1a	non-LTR	FBgn0193895	Gr28a	CDS
SFS	Scf_2L	10673070	Cr1a	non-LTR	FBgn0193610;FBgn0043403	Samuel;Acp32	CDS
SFS	Scf_2L	11487237	Doc2-element	non-LTR	FBgn0193565;FBgn0195164	Pde1c;Mal	CDS
SFS	Scf_2L	17472390	Rt1b	non-LTR	FBgn0193269;FBgn0195475	btv;CG5674	CDS

SFS	Scf_2L	17484113	Max-element	LTR	FBgn0193269	btv	CDS
SFS	Scf_2R	4105774	Cr1a	non-LTR	FBgn0182038	Gr43a	CDS
SFS	Scf_2R	4106284	Rt1c	non-LTR	FBgn0182038	Gr43a	CDS
SFS	Scf_2R	9422192	GATE	LTR	FBgn0045629	CG17575	CDS
SFS	Scf_2R	9422316	diver2	LTR	FBgn0045629	CG17575	CDS
SFS	Scf_2R	12643581	TART	non-LTR	FBgn0196857	Dg	CDS
SFS	Scf_2R	12644408	Max-element	LTR	FBgn0196857	Dg	CDS
SFS	Scf_2R	12682578	Cr1a	non-LTR	FBgn0182918	spin	CDS
SFS	Scf_2R	12687273	Tc1	TIR	FBgn0182918;FBgn0270363	spin	CDS
SFS	Scf_2R	13767755	diver2	LTR	FBgn0183008;FBgn0269733	mbl	CDS
SFS	Scf_2R	18003904	1731	LTR	FBgn0270272;FBgn0196498	CG10433	CDS
SFS	Scf_2R	21400982	297	LTR	FBgn0196212	lov	CDS
SFS	Scf_3L	5133514	Cr1a	non-LTR	FBgn0184928;FBgn0270213	shep	CDS
SFS	Scf_3L	6582905	Cr1a	non-LTR	FBgn0184837	ple	CDS
SFS	Scf_3L	9187744	rooA	LTR	FBgn0184651;FBgn0182438	CG3967;aay	CDS
SFS	Scf_3L	14764140	I-element	non-LTR	FBgn0184300	CG12316	CDS
SFS	Scf_3L	14765285	diver2	LTR	FBgn0184300	CG12316	CDS
SFS	Scf_3L	16192172	Max-element	LTR	FBgn0012901	tra	CDS
SFS	Scf_3R	817473	TART	non-LTR	FBgn0191132	corto	CDS
SFS	Scf_3R	819767	P-element	TIR	FBgn0191132	corto	CDS
SFS	Scf_3R	2852822	Cr1a	non-LTR	FBgn0191002;FBgn0269480	Alh	CDS
SFS	Scf_3R	2853647	diver2	LTR	FBgn0191002;FBgn0269480	Alh	CDS
SFS	Scf_3R	5341809	412	LTR	FBgn0269512	Gr92a	CDS
SFS	Scf_3R	9047181	G2	non-LTR	FBgn0191767	ss	CDS
SFS	Scf_3R	12841828	GATE	LTR	FBgn0027803	Desat1	CDS
SFS	Scf_3R	13890061	TART	non-LTR	FBgn0025030	pros	CDS
SFS	Scf_3R	13891467	TART	non-LTR	FBgn0025030	pros	CDS
SFS	Scf_3R	13918033	GATE	LTR	FBgn0025030;FBgn0192130;	pros;KP78a;KP	CDS
SFS	Scf_3R	13919859	Cr1a	non-LTR	FBgn0025030;FBgn0192130;	pros;KP78a;KP	CDS
SFS	Scf_3R	17194983	Cr1a	non-LTR	FBgn0192358	Gr93a	CDS
SFS	Scf_3R	19075663	Cr1a	non-LTR	FBgn0189891	prt	CDS
SFS	Scf_3R	20049788	GATE	LTR	FBgn0192574	slo	CDS
SFS	Scf_3R	23983700	hobo	TIR	FBgn0192817;FBgn0192816	CheB98a;intr	CDS
SFS	Scf_X	2150649	Transpac	LTR	FBgn0187984	tko	CDS
SFS	Scf_X	5031359	Cr1a	non-LTR	FBgn0187886	frma	CDS
SFS	Scf_X	7482466	297	LTR	FBgn0068641	Tbh	CDS
SFS	Scf_X	7999381	Cr1a	non-LTR	FBgn0188488	Nrg	CDS
SFS	Scf_X	18638406	P-element	TIR	FBgn0270786;FBgn0268400	Hers;amn	CDS
SFS	Scf_X	18645589	transib2	TIR	FBgn0270786;FBgn0268400	Hers;amn	CDS
SFS	Scf_X	18648483	P-element	TIR	FBgn0270786;FBgn0268400	Hers;amn	CDS
SFS	Scf_X	18648695	P-element	TIR	FBgn0270786;FBgn0268400	Hers;amn	CDS

Fig. S1

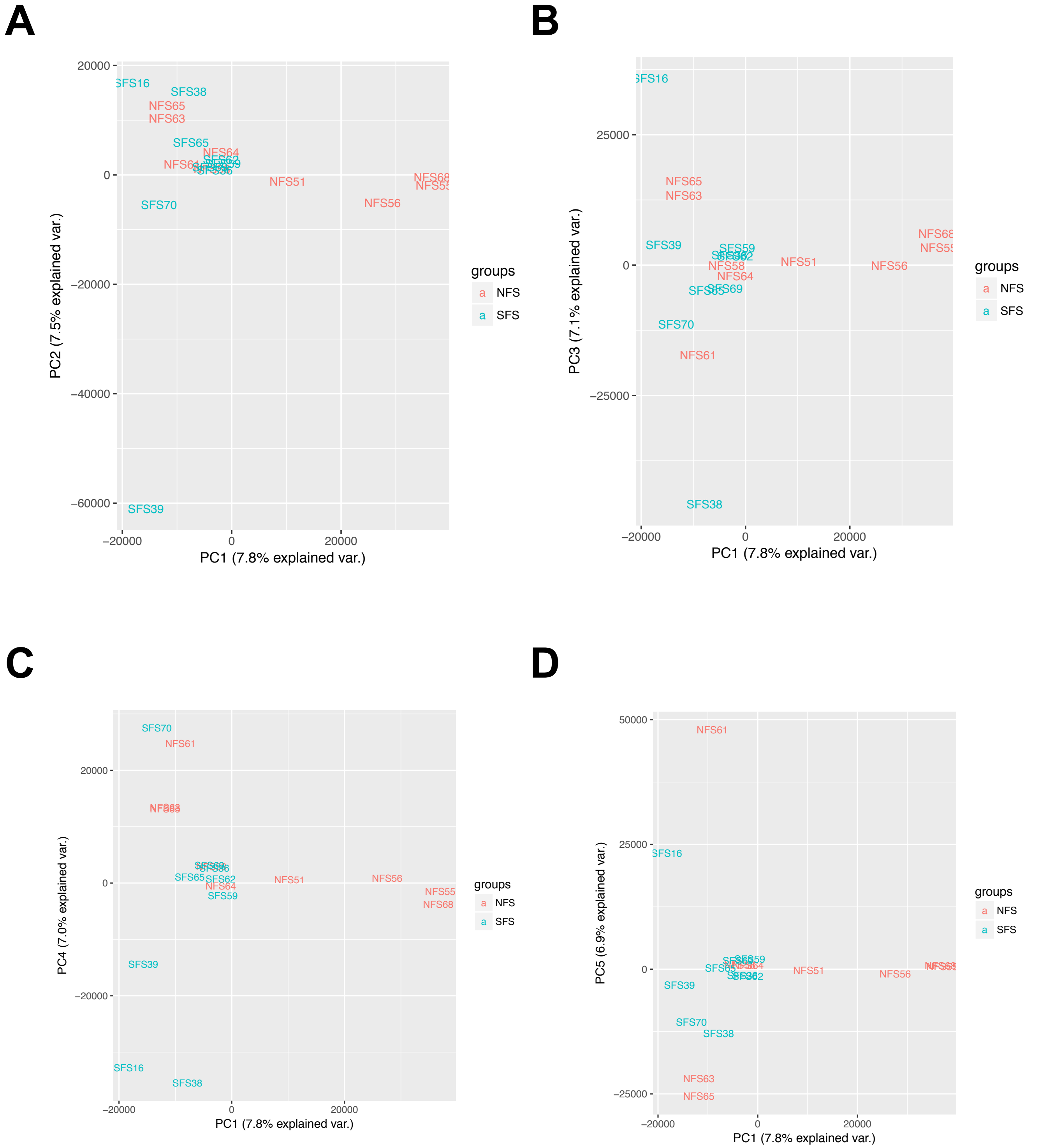
A
B
C
D
PC1 (7.8% explained var.)
PC2 (7.5% explained var.)
PC3 (7.1% explained var.)
PC4 (7.0% explained var.)
PC5 (6.9% explained var.)
groups
NFS
SFS
SFS16
SFS38
NFS65
NFS63
SFS65
NFS64
NFS51
NFS68
NFS56
SFS70
SFS39
SFS59
NFS58
SFS69
NFS61
NFS55
SFS62
SFS36
20000
0
−20000
−40000
−60000
25000
−25000
50000

Fig. S2

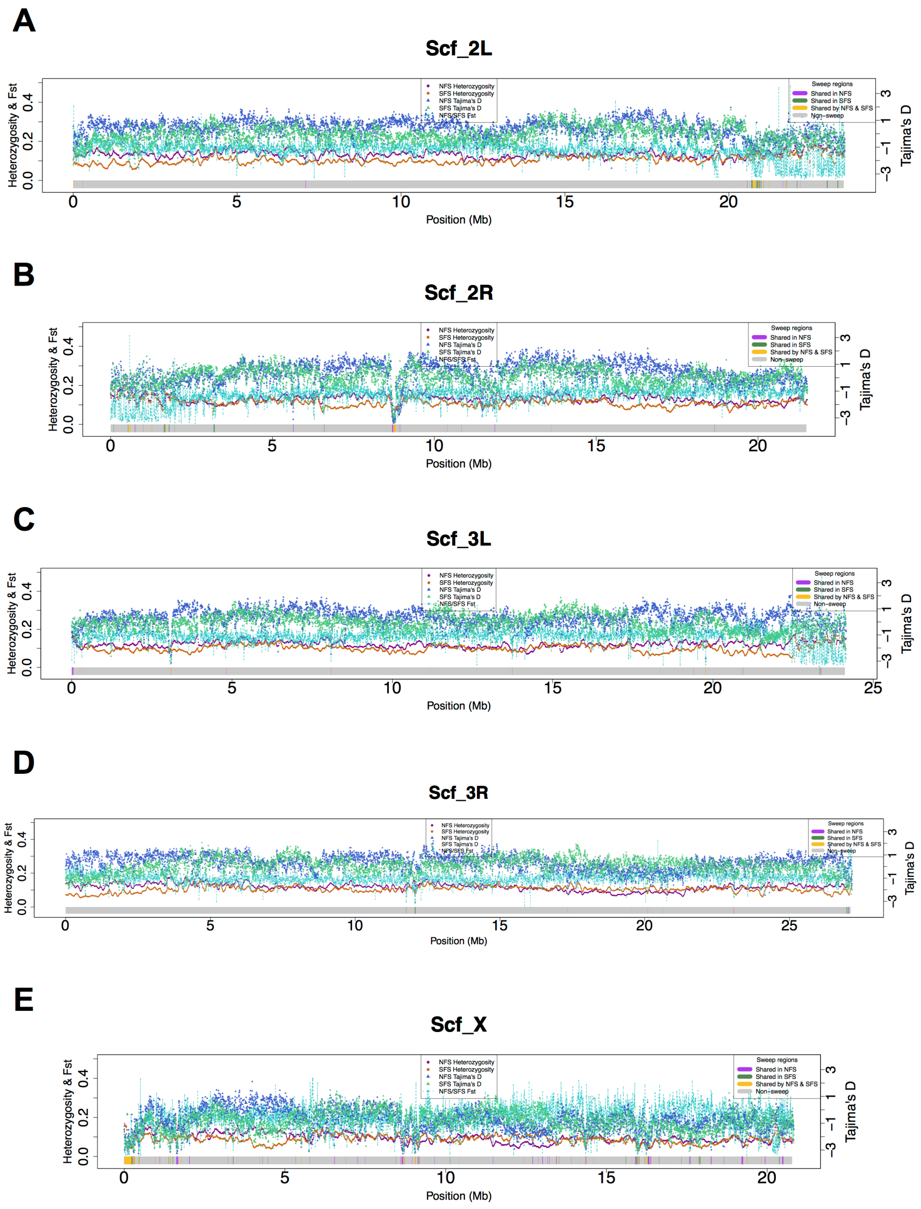
A
Scf_2L
Heterozygosity & Fst
Tajima's D
Position (Mb)
NFS Heterozygosity
SFS Heterozygosity
NFS Tajima's D
SFS Tajima's D
NFS/SFS Fst
Sweep regions
Shared in NFS
Shared in SFS
Shared by NFS & SFS
Non−sweep
B
Scf_2R
C
Scf_3L
D
Scf_3R
E
Scf_X

Fig. S3

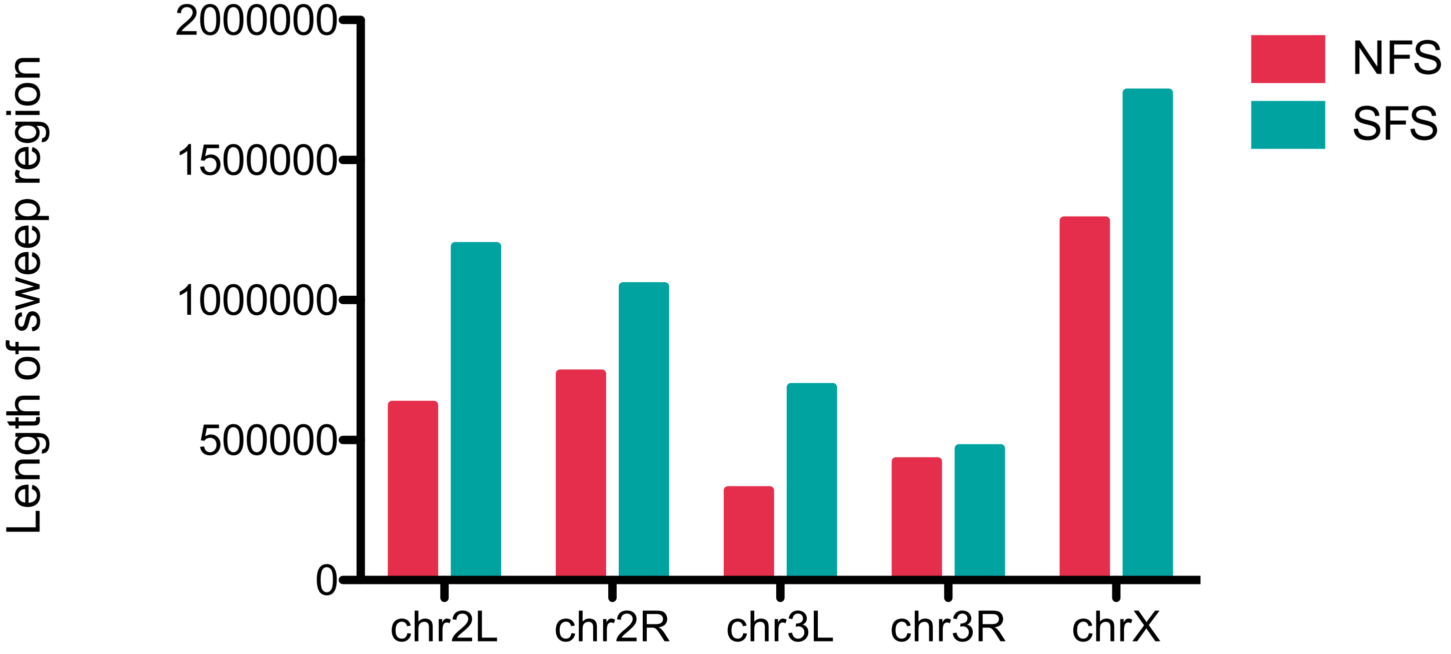

2000000
1500000
1000000
500000
0
Length of sweep region
chr2L
chr2R
chr3L
chr3R
chrX
NFS
SFS

Fig. S4

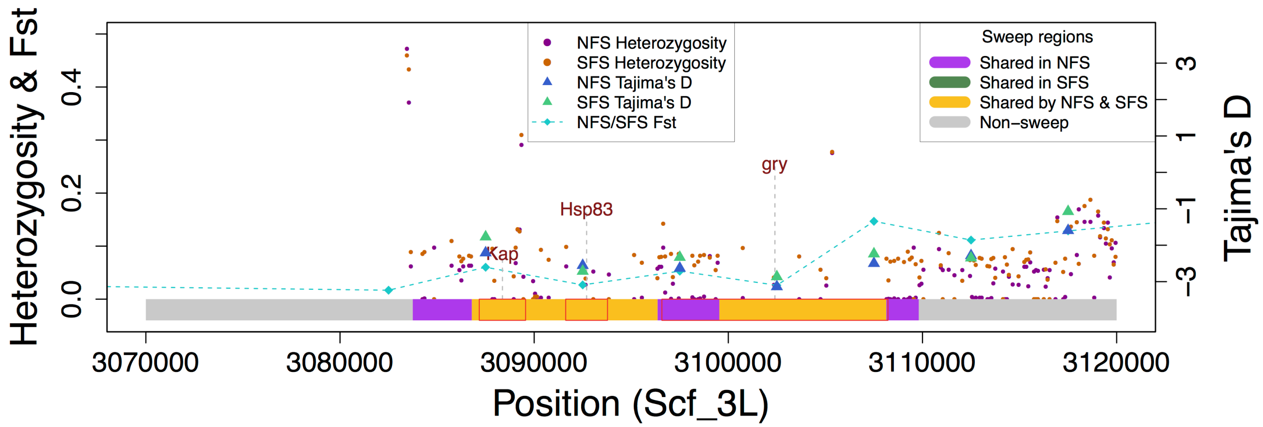
NFS Heterozygosity
SFS Heterozygosity
NFS Tajima's D
SFS Tajima's D
NFS/SFS Fst
Sweep regions
Shared in NFS
Shared in SFS
Shared by NFS & SFS
Non−sweep
Kap
Hsp83
gry
Heterozygosity & Fst
0.0
0.2
0.4
Tajima's D
−3
−1
1
3
3070000
3080000
3090000
3100000
3110000
3120000
Position (Scf_3L)

Fig. S5

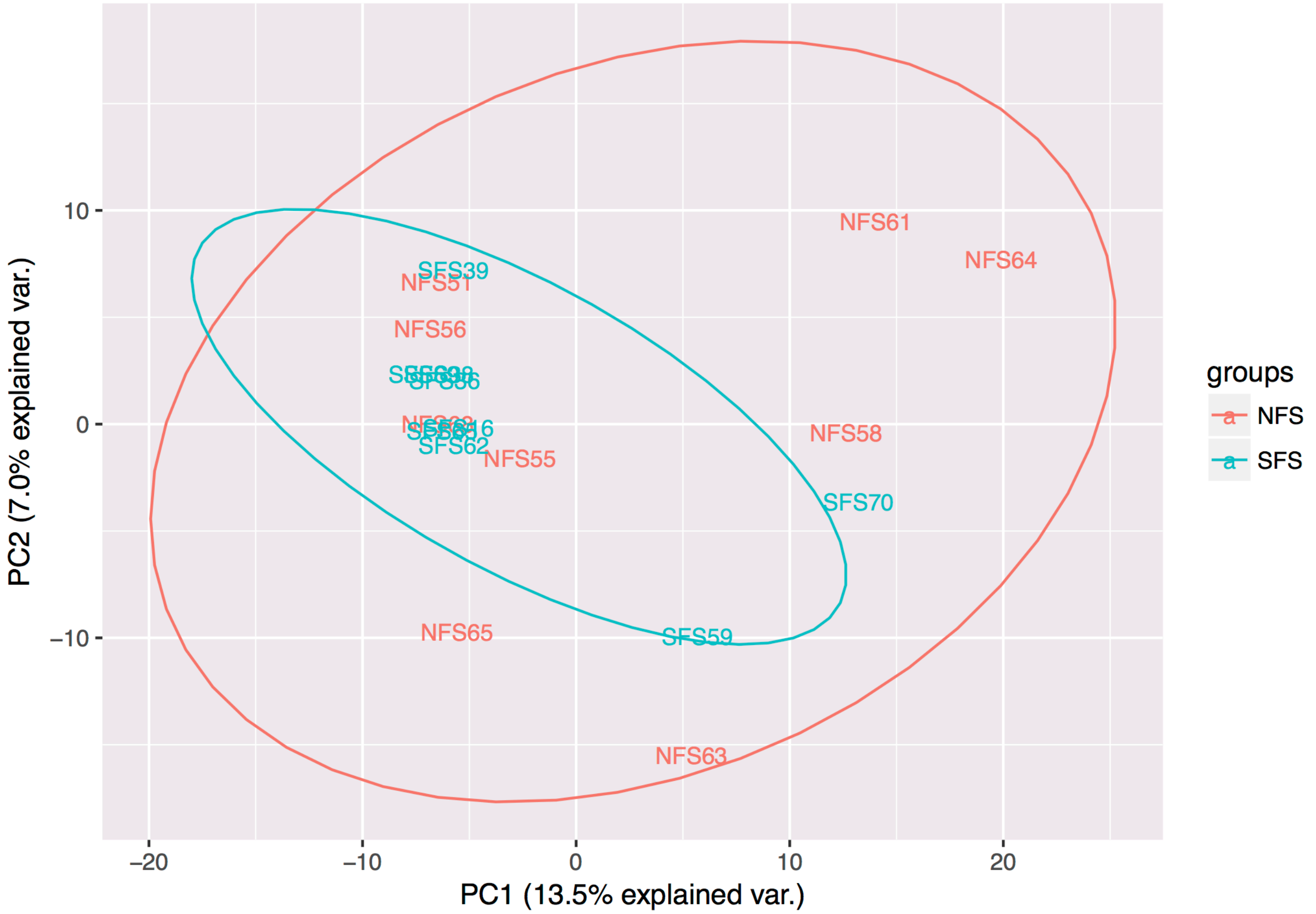

PC1 (13.5% explained var.)
PC2 (7.0% explained var.)
groups
NFS
SFS
NFS61
NFS64
SFS39
NFS51
NFS56
NFS58
NFS55
SFS70
NFS65
SFS59
NFS63
SFS62
